# Ring Ultramicroelectrodes for Current-Blockade Particle-Impact Electrochemistry

**DOI:** 10.1021/acs.analchem.2c01503

**Published:** 2022-07-06

**Authors:** Taghi Moazzenzade, Tieme Walstra, Xiaojun Yang, Jurriaan Huskens, Serge G. Lemay

**Affiliations:** MESA+ Institute and Faculty of Science and Technology, University of Twente, P.O. Box 217, 7500 AE Enschede, The Netherlands

## Abstract

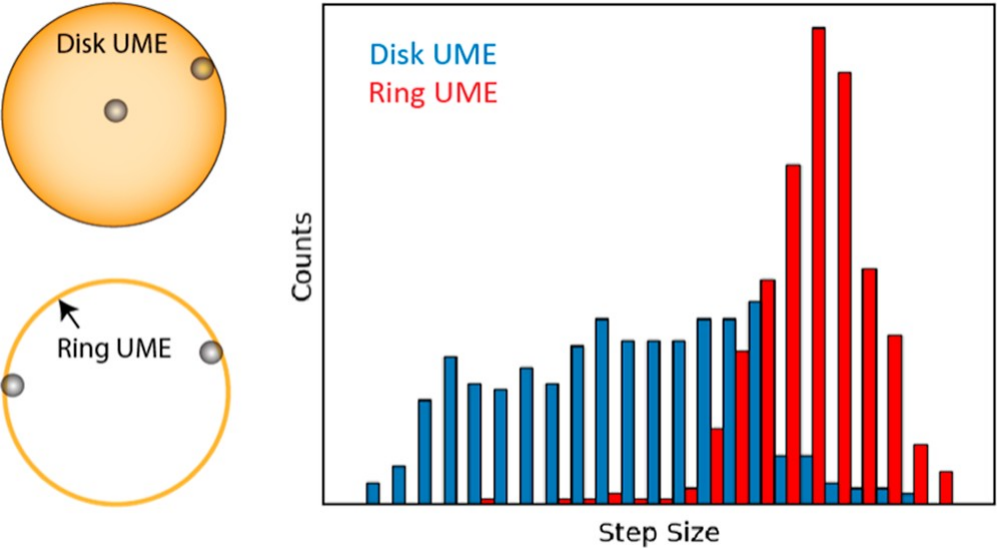

In current-blockade impact electrochemistry, insulating particles are detected amperometrically as they impinge upon a micro- or nanoelectrode via a decrease in the faradaic current caused by a redox mediator. A limit of the method is that analytes of a given size yield a broad distribution of response amplitudes due to the inhomogeneities of the mediator flux at the electrode surface. Here, we overcome this limitation by introducing microfabricated ring-shaped electrodes with a width that is significantly smaller than the size of the target particles. We show that the relative step size is somewhat larger and exhibits a narrower distribution than at a conventional ultramicroelectrode of equal diameter.

## Introduction

Particle-impact electrochemistry is a set of techniques, in which individual micro- and nanoscale entities are detected in real time as they impinge upon a miniaturized electrode. Depending on the electroactivity of the particles and of the electrode, particle collisions lead to various types of stochastic, discrete signatures in amperometric measurements. Information on the particles such as surface properties, catalytic activity, size, and even shape can be inferred from these measurements,^1−12^ also in optically opaque solutions.^13^ Impact methods have also been used for detecting individual biomolecules^14−18^ and have been suggested as candidate single-entity electrochemical transducers for digital biosensing.^19^

**Figure 1:**
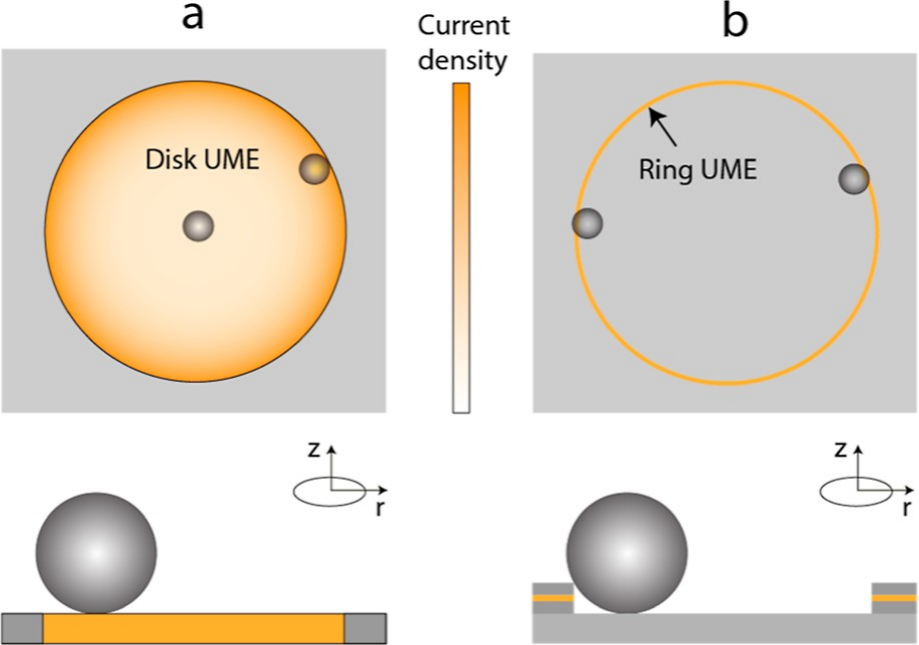
Schematic representation of disk and ring UMEs. (a) For the disk UME, the region near the rim of the disk has the highest mediator flux and correspondingly the highest current density. The current density (given by eq 3) is minimal at the center of the electrode, but with a large region surrounding the center, where it is nearly constant. Because of the variations in current density, particles landing near the center or the edge of the disk lead to different signal sizes. (b) For the ring UME, the mediator flux and the current density are uniform along the circumference. Variations across the thickness of the ring do occur, but on such a small length scale that they are averaged over in a particle-impact experiment. This leads to consistent current steps. Because migration usually dominates transport of the particles in impact experiments, the particles are attracted to the ring and remain confined in the cavity.

An early variant of particle-impact electrochemistry is current blockade. Here, insulating particles are detected as a decrease in an otherwise steady-state amperometric signal as they interfere with the mass transport of a redox mediator to the electrode.^20^ In addition to inert synthetic particles, this method has been employed to detect bacteria,^21^ vesicles,^22^ viruses,^17^ and biomolecules.^15^ It has further been employed as a tool for characterizing the size of biomacromolecules^15^ and graphene oxide sheets.^23^ A limitation of the method, however, arises because the size of the steps in the measured current depends on the location of the particle on the electrode.^24,25^ This occurs because the mediator flux is not uniform over the surface of a planar electrode, as illustrated in Figure 1a. For a disk ultramicroelectrode (UME), for example, the current density is highest near the rim of the electrode and smallest at its center.^26^ This inhomogeneity causes two main problems in particle blockade measurements with disk-shaped UMEs: (1) false-positive events introduce error in particle-counting experiments^24,25^ and (2) biases are introduced in particle-sizing experiments that are based on the amplitude of the current-blockade steps.^24,25^

False-positive detection events are the first consequence of the inhomogeneous flux of redox mediators on disk UMEs. In most blockade impact experiments, migration is the dominant mode of transport for charged particles, while the flux of the inert redox mediator remains diffusion-limited.^1^ The disk edge exhibits a higher flux density and, consequently, a stronger residual electric field. Hence, charged particles tend to migrate toward the edge of the UME. Furthermore, as a result of this *electrophoretic edge effect*, particles that are adsorbed near the center of the electrode subsequently migrate toward the edge. This dynamic rearrangement of particles leads to unwanted steps that result in overestimates during particle counting measurements.^24,25^

The second issue is the uneven signal that is due to the *mediator edge effect*. Due to the non-uniform mediator flux, the location where particles land on the surface can cause uneven current step sizes, with particle collisions near the edge of the disk leading to bigger steps than near the center. This occurs independently of whether particles are transported primarily by migration or diffusion. This issue complicates signal analysis in blockade impact measurements, and in particular renders particle size measurements difficult. In blockade impact, particle size (radius, *r*_Δ*i*_) can be estimated from the current step magnitude using , where *i*_ss_ is the steady-state current, Δ*i*_ss_ is the current step magnitude, *a* is the radius of the UME, and α is a numerical factor that depends on the electrode geometry.^15^ This expression, however, neglects the variability in the step size introduced by the mediator edge effect.^25^ Uneven current steps can also indirectly affect particle-counting measurements if collisions near the disk center lead to steps that are smaller than the background noise, while collisions at the edge cause measurable steps. This limits the useable electrode-to-particle size ratio.^27^ Finally, a variable step size is particularly problematic if a particular step size Δ*i*_ss_ is used as the signature of a specific event such as in biorecognition.^28^

Because the discrepancies in the signal size and frequency both stem from the inhomogeneous flux of the redox mediator, solving this issue can significantly improve the accuracy of blockade impact measurements. Recently, Renault et al. demonstrated a way to circumvent non-uniform flux by using a hemispherical electrode, for which the current density is uniform over the surface of the electrode.^29^ This uniformity stems from the spherical symmetry of this geometry, at least under conditions, where surface conduction and electrokinetic effects caused by the insulating shroud surrounding the electrode can be neglected. In this work, the electrodes were realized using liquid Hg. While it serves as an important proof of concept, this system remains somewhat impractical for routine measurements due to the additional fluid handling and safety considerations attached to Hg.

Here, we introduce an alternative electrode geometry to overcome the non-uniform flux of redox mediators in blockade impact electrochemistry. The electrodes, which are fabricated using optical lithography, consist of a ring that is thinner than the particles to be detected. The structure is analogous to ring electrodes in some ring-disk electrode pairs used for redox cycling and generator-collector experiments.^30,31^ This approach avoids the problems caused by an inhomogeneous current density. Particles colliding at any position along the circumference of the ring encounter a similar environment. Furthermore, while the mediator flux does vary across the width of the ring, the larger size of the particles ensures that the measured signal includes contributions from the entire distribution of fluxes (Figure 1b), largely eliminating positional effects. In a migration-limited regime, particles remain confined to the contour of the ring through the electric field and block a similar amount of redox molecules. Furthermore, this geometry is fully compatible with planar microfabrication methods, unlike a hemispherical geometry, while also allowing impact measurements at current densities higher than those encountered at disk UMEs of the same diameter. We show experimentally that ring electrodes exhibit a narrower distribution of step sizes and a higher relative size sensitivity when compared to data for disk electrodes of the same diameter.

## Experimental Section

### Chemicals and Instrumentation

In this study, we employed 1,1′-ferrocenedimethanol (Sigma-Aldrich, 372625) as the redox mediator and KCl (Sigma-Aldrich, P9333) as the supporting electrolyte. All the measurements were performed using 1 μm diameter (standard deviation = 0.03 μm) polystyrene beads (Polysciences, 07310). The zeta potential of the polystyrene beads was measured in working solution (0.67 mM ferrocenedimethanol and 7.5 mM KCl) using a Zetasizer Nano ZS (Malvern Panalytical). The 10 μm disk platinum UME was purchased from BASi (BASi/MF-2005). The Pt disk was polished with alumina slurry (1, 0.3, and 0.05 μm, Buehler, Lake Bluff, U.S.A). Milli-Q water with 18.2 MΩ·cm^–1^ resistivity (Milli-Q Advantage A10) was used for preparing all the solutions.

### Device Fabrication

**Figure 2:**
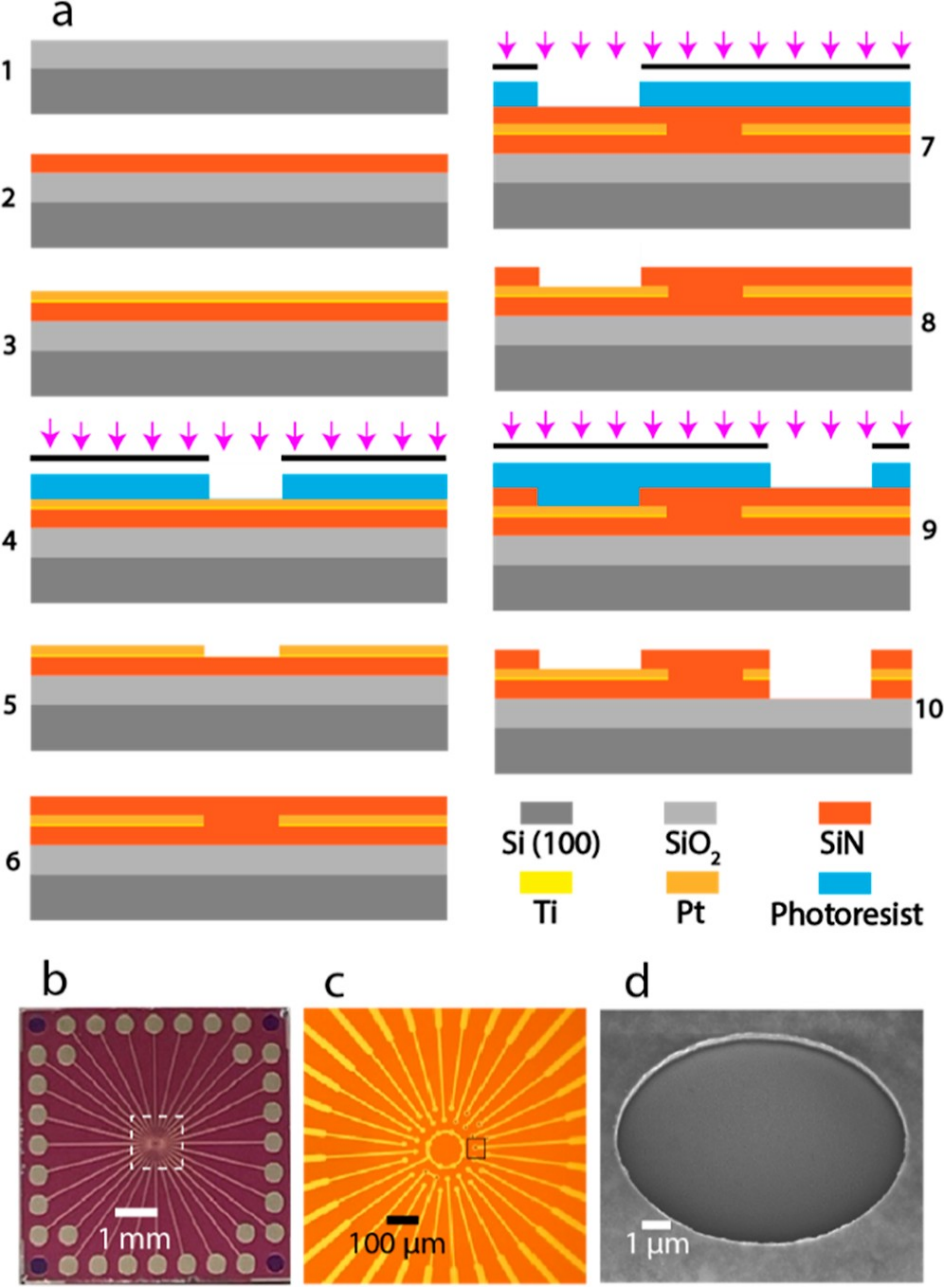
(a) Schematic representation of cross section at different steps of the process flow for the fabrication of disk and ring UMEs: 1—wet oxidation, 2—SiN deposition by PECVD, 3—sputtering of Ti and Pt via T’COathy, 4—lithography (alignment and exposure) and development of the photoresist, 5—etching Pt and Ti layers via IBE and stripping of the photoresist, 6—SiN deposition via PECVD, 7—lithography and development of the photoresist, 8—etching SiN layer via RIE (exposing the disk UMEs and external connections) and stripping of the photoresist, 9—lithography and development of the photoresist, and 10—etching SiN/Pt–Ti/SiN layers using a combination of RIE/IBE/RIE methods (exposing the ring UMEs) and stripping of the photoresist. (b) Optical microscopy image (top view, scale bar 1 mm) of the fabricated device. (c) Optical microscopy image (top view, scale bar 100 μm) of the fabricated device showing interconnects and electrodes of different sizes at the center. (d) Scanning electron microscopy (SEM) image of a ring UME with diameter of 10 μm and a thickness of 50 nm from a 45° viewing angle (scale bar 1 μm). Pt appears as a bright ring.

Ring and disk UMEs with different diameter were fabricated on the same chips using a combination of thin-film deposition, optical lithography, and etching procedures. Figure 2a represents the fabrication process flow schematically; the steps described below correspond to the numbered sub-panels in Figure 2a. The full process flow is also illustrated in Figure S1.

A one-side polished (OSP) silicon wafer (⟨100⟩, boron-doped p-type) with a thickness of 525 ± 25 μm was used as the substrate. Wet oxidation of silicon was performed to grow a 500 nm SiO_2_ layer (step 1). A 100 nm silicon nitride (SiN) layer was then deposited via PECVD (step 2). In step 3, 5 nm titanium (Ti) as the adhesion layer and 50 nm platinum (Pt) were sputtered using an ion-beam system (home-built T’COathy system, MESA + NanoLab, the Netherlands). Standard optical lithography was then performed to define the geometry of the metal electrodes as well as connecting wires and pads for external connections (step 4). After developing the resist, in step 5, the 50/5 nm Pt/Ti layers were etched via ion beam etching (IBE, Oxfordi300Plus), and the photoresist was stripped with O_2_ plasma (TePla300, PAV TePla AG, Germany). In step 6, the second layer of SiN (100 nm) was deposited on the surface via PECVD. In step 7, standard lithography was again performed to expose disk electrodes and mm-scale connections to the device. Then, the exposed SiN layer was etched via reactive ion etching (RIE) with CHF_3_/O_2_ plasma (home-built TEtske system, MESA+ NanoLab, the Netherlands), and the photoresist was stripped with TePla300 (step 8). In order to create the ring electrodes, standard lithography was again performed as per the above (step 9). The exposed circular areas with diameters of 2.5, 5, and 10 μm were etched using a combination of RIE/IBE/RIE to etch the SiN/Pt/SiN layers, respectively (step 10), and the photoresist was stripped with O_2_ plasma. Due to the slow etch rate of the RIE for Pt, IBE was used for etching this layer. Complete etching of the bottom layer of SiN was monitored using optical microscopy; exposure of SiO_2_ in the 10 μm diameter wells led to a color change. The wafer was coated with photoresist before dicing to prevent contamination during dicing.

### Electrochemical Measurements

The measurements were performed using a two-electrode configuration in a custom Faraday cage. No auxiliary electrode was required as the current levels remained on the order of ∼1 nA or smaller. A constant potential of +0.35 V was applied to a microfabricated Pt ring or a commercial Pt disk (BASi/MF-2005) working electrode with respect to the Pt pseudoreference electrode wire (0.20 mm diameter, ∼4 mm length exposed to solution), corresponding to an oxidizing overpotential. Data for the commercial UME are shown here to facilitate comparison to other works, but the on-chip disk UMEs yielded equivalent results. A transimpedance amplifier (Femto, DDPCA-300, GmbH, Berlin, Germany) and a homemade Labview (v2013) program were used to monitor the current. The measurements were performed using 1 μm diameter polystyrene particles with a density of 4.55 × 10^10^ particles/mL (stock solution). The particle solution was diluted 500 times in 0.67 mM ferrocenedimethanol as the redox mediator (prepared in Milli-Q water) as the working solution for the measurements. We further employed KCl as the supporting electrolyte with a concentration of 7.5 mM in all the measurements. The UME was polished mechanically in a figure-eight motion with alumina particles with sizes of 1.0, 0.3, and 0.05 μm polish, rinsing the electrode between the different polishing steps. For the microfabricated devices, individual diced chips were successively cleaned with acetone, Milli-Q water, and isopropyl alcohol and sonication for 5 min for each step so as to remove a protective photoresist protective layer introduced for dicing. The chips were placed in a homemade stage including a custom socket for making connections [36-pin Land Grid Array (LGA) package, 0.70 mm pitch, Interconnect Devices (IDI)]. A polydimethylsiloxane cylinder was used as the cell, and the pseudoreference electrode was inserted through the top opening. The experimental geometry is illustrated in Figure S2.

### Numerical Simulations

In order to obtain a more quantitative assessment of the current density on disk UMEs, finite-element calculations of the diffusive mass transport of the redox mediator to three-dimensional models of the electrode were performed. These finite-element numerical simulations were performed with COMSOL Multiphysics 5.3a (Supporting Information). The step size was calculated by subtraction of the steady-state current with and without the particle on the surface.

### Analysis of Experimental Current Steps

Due to the dependency of the step size on the baseline current, the current step sizes (Δ*i*_ss_) were normalized to the steady-state current (*i*_ss_) immediately before each step, and the Δ*i*_ss_/*i*_ss_ ratio was used to compare the step sizes in the two geometries. Several measurements were performed to generate sufficient numbers of steps for the statistical analysis. The total numbers of steps were 476 for both the ring and disk UMEs. In order to limit the influence of previously landed particles on the step size, a limited number of collisions (at most 15 collisions from each measurement) were used in the analysis. Using fewer collisions per experiment did not modify the shape of the histograms apart from introducing more statistical noise (Supporting Information). Due to the decrease in the steady-state current, which is particularly pronounced in the first seconds of the measurements, the steps were counted from the second step to step number 16. The step size analysis was performed using a custom script. First, the amperometric measurements were filtered using a Savitzky–Golay filter to remove the high-frequency noise. Each smoothed curve was then differentiated, and the location of the steps was identified as sharp spikes in the resulting curve (see the Supporting Information for details).

## Results and Discussion

Our strategy for obtaining consistent signals in current blockade measurements is to create a ring electrode that is smaller in width than the target particles to be detected, so that each particle-impact event is geometrically equivalent regardless of where it occurs along the circumference of the electrode. For micron-sized particles or smaller, this implies nanoscale ring widths. While electron-beam lithography is a good approach for the fabrication of planar electrodes with such dimensions, it is not widely available and does not lend itself well to large-scale production. In this report, we instead focus on standard, widely available optical lithography techniques. To control the width, we rely on the ability to control the deposition of thin films very accurately. As a consequence of this, the ring electrode is embedded in the wall of a shallow cavity rather than being fully planar. The thickness of the ring is determined by the amount of metal deposited. Although the fabricated device in this report could be used for analyzing smaller particles, 1 μm particles were employed to be comparable with the results of a conventional 10 μm disk UME experiment. As illustrated schematically in Figure 1b, the particles are bigger than the height of the SiN/Pt/SiN well. As a consequence, there is no direct contact between the particle and the electrode surface, and the landed particles are confined in the corner of the ring by the electric field.

We first present measurements on disk electrodes and analyze the step size distribution in order to provide a baseline against which the ring electrodes can be compared.

### Disk UME Measurements

**Figure 3:**
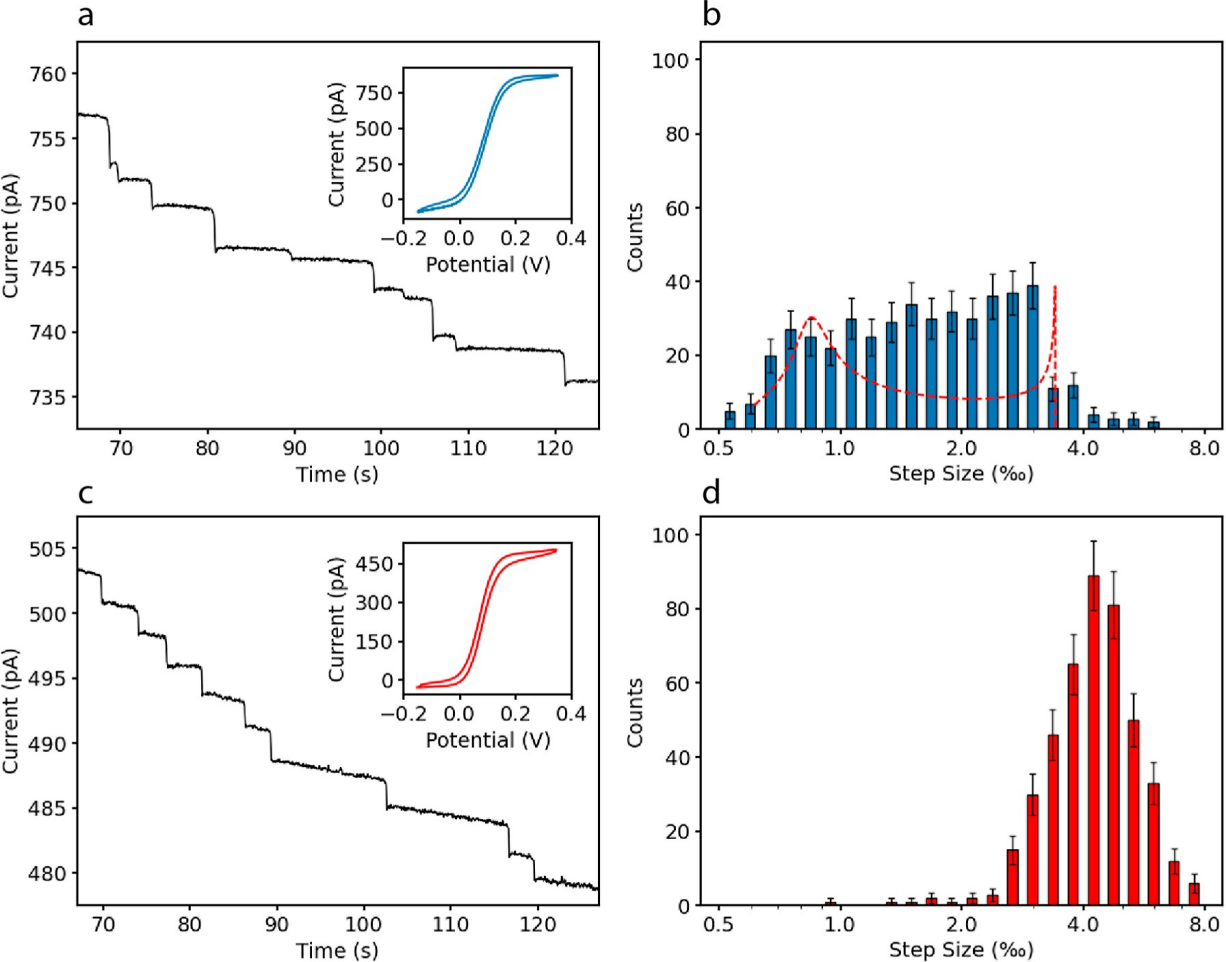
(a) Current–time response of blockade impact measurement with 10 μm disk UME, and 1 μm negatively charged particles in 0.67 mM ferrocenedimethanol solution exhibits uneven current steps with a range from ≈1 to 4 pA. (b) Histogram of the step sizes for 476 current steps obtained in different measurements. The red dashed curve represents the distribution obtained from eq 4 with no fitting parameter. (c) Chronoamperograms and CV of the measurements with the fabricated recessed ring electrodes with 10 μm diameter and 50 nm thickness. (d) Histogram of the step sizes for the 10 μm ring. The *x*-axes in (b,d) show the step size per mille (‰) on a logarithmic scale (the data are also shown on a linear scale in the Supporting Information). The step size is normalized by the current immediately before each step. This facilitates comparison with previous reports with different geometries and measurements at different redox mediator concentrations.^29^

The diffusion-limited current to a shrouded disk electrode is given by^26^
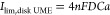
1Here, *n* is the number of electrons, *F* is the Faraday constant, *D* is the diffusion coefficient of the redox species at concentration *C*, and *a* is the radius of the disk. A diffusion-limited steady-state current of ≈850 pA was obtained experimentally (Figure 3a), which is in good agreement with the calculated value using eq 1 (*I*_theoretical_ = 860 pA, *D* = 6.7 × 10^–10^ m^2^·s^–1^).

For the negatively charged 1 μm particles, the supporting electrolyte concentration employed here results in migration-limited transport. This is in contrast to transport of the redox mediator, which is diffusion-limited. This regime of transport was chosen to ensure multiple discrete events per measurement over a practical time scale. However, the supporting electrolyte ratio was sufficiently high (>10) to suppress electroosmotic flows (EOF). This avoided the possibility that EOF could repel the colliding particles from the electrode along the surface, which would lead to current spikes instead of well-defined steps and would complicate interpretation.^32,33^ This regime enables measurement of particles at ultralow concentrations, decreasing the probability of particle adsorption on UME surfaces before starting the measurements and the probability of particles cocollision during the measurements.

As can be seen in the raw data of Figure 3a, the current–time response of blockade impact of 1 μm particles on a 10 μm disk UME exhibits steps with uneven sizes with a range from approximately 1–4 pA. A histogram of the normalized magnitude of the steps is shown in Figure 3b, illustrating the broad distribution of step sizes in measurements with disk UMEs. This has previously been explained as resulting from the inhomogeneous current distribution at the surface (mediator edge effect).^20,24,25,29^ This reasoning can be further formalized as follows. We define the area of the electrode with a radius between *r* and *r* + d*r*, where d*r* is an infinitesimal increment, as *g*_*r*_(*r*)d*r* = 2π*r* d*r*. We similarly define the distribution of current step sizes, *g*_Δ*i*_(Δ*i*)d(Δ*i*), as the relative number of sites on the electrode, where the step size falls in the range Δ*i* to Δ*i* + d(Δ*i*). Δ*i* is a function of *r* because of the non-uniform mediator flux density. Equating *g*_*r*_(*r*)d*r* = *g*_Δ*i*_(Δ*i*)d(Δ*i*) yields
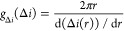
2

The probability that a particular step has an amplitude Δ*i* is proportional to *g*_Δ*i*_(Δ*i*), because this dictates the number of sites that yield this value of Δ*i*, times the particle flux to that region. Noting that the mediator current density *j*(*r*) at a disk electrode as a function of radial position *r* has the form^34^
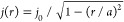
3and that the migrational transport of particles resulting from the residual ohmic electric field approximates the same form, we have for the expected probability density of step sizes
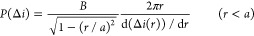
4Here, *B* is a normalization factor independent of *r*. In this simple model *P*(Δ*i*) = 0 for *r* > *a* because there is no electric field driving the particles to the insulating shroud; however, note that this simplification would need to be lifted for situations, where diffusion contributes substantially to particle mass transport.

In order to evaluate eq 4 for *P*(Δ*i*), it is necessary to have an expression for the dependence of the step size Δ*i*(*r*) on *r*. We evaluated this function using finite-element methods, as detailed further in the Supporting Information. The final predicted distribution, which does not include any free parameter, is shown in Figure 3b. It exhibits two peaks. The peak at high step sizes corresponds to particles landing near the edge; although this corresponds to a small area, the collision density is high due to the stronger electric field caused by the higher diffusive flux of redox mediator in this region. The peak at low step sizes corresponds to collisions in the central area of the disk with a larger area, but a lower current density and electric field. While there is good agreement between the predicted and measured range of current step sizes, the sharp peaks in the distribution are not observed in the experiment. We attribute this additional broadening to elements missing in the model including residual EOFs and the influence of the finite particle size on migration in a non-uniform electric field.

### Ring UME Measurements

The geometry of the microfabricated ring UMEs is shown in Figure 2. The ring electrodes were positioned in a shallow cylindrical cavity with the electrode located on the side wall of the cavity. The current to a thin planar ring (in the limit, where the width of the ring Δ is much smaller than the ring radius ρ, Δ ≪ ρ) is
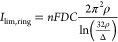
5

While our geometry is more complex, this expression serves as a first approximation to estimate the current levels that can be expected. The ratio of this current and of that at a disk with the same radius (*a* = ρ) is *I*_lim,ring_/*I*_lim,disk_ ≈ π^2^/2ln(32ρ/Δ). The slow inverse logarithmic dependence of this ratio on the ring width, Δ, ensures that even a thin ring can exhibit total currents of the same order of magnitude as a disk UME of the corresponding diameter. In particular, eq 5 predicts a ratio *I*_lim,ring_/*I*_lim,disk_ ≈ 0.6 for our 10 μm diameter devices, which is remarkable considering that the electrode area is a factor ≈50 times smaller for the ring.

The measurements with ring UMEs were performed under the same conditions as the disk measurements. The voltammogram of the fabricated 10 μm diameter and 50 nm thick ring UME exhibits a typical sigmoidal shape for diffusion-limited transport (inset, Figure 3c). A limiting current of ≈500 pA was obtained. This corresponds to *I*_lim,ring_/*I*_lim,disk_ = 0.59, in good agreement with the theoretical estimate.

Figure 3c shows the amperometric response of a 10 μm diameter ring UME during a current blockade measurement. Qualitatively, the response exhibits relatively uniform step sizes. The frequency of collisions is comparable to that for the disk electrode, consistent with the similar magnitude of the current. Figure 3d shows the corresponding histogram of step sizes. The ring UME results in a single, relatively narrow, peak representing uniform current step sizes. This confirms the expectation of a more uniform current density along the perimeter of the ring, in such a way that collision of the particles anywhere on the ring blocks a similar amount of redox mediator.

More quantitatively, the standard deviations of the step size for the disk electrode (blue histogram) and ring electrode (red histogram) are 0.25 and 0.13, respectively. In addition to the uniformity of the signals, ring UMEs exhibit larger relative step sizes. These two observations translate into a higher relative step size sensitivity for rings under identical conditions.

The histogram in Figure 3d nevertheless exhibits a significant width. One might try to explain this broad histogram as resulting from particles landing close to each other after multiple collisions. If this were the case, analyzing a lower number of steps per measurement should result in a narrower distribution. However, the histogram shape and standard deviation of the step size remain nearly unchanged in the ring UME when analyzing the first 10, 7, or 5 steps of the same measurements (Figure S5), ruling out this mechanism.

The breadth of the histograms could also be due to non-idealities in the experiment. One possibility is material redeposition in the inner wall of the cavity in the last etching steps leading to a non-uniform metal thickness. This non-uniformity can be improved in the future by implementing alternative approaches for etching the SiN/Pt/SiN stack. Material redeposition as well as an uneven etch depth inside the ring may also lead to different particle heights when adsorbing inside the ring. This can be mitigated in the future by etching deeper so that the particles adsorb onto the electrode ring itself without touching the bottom of the cavity. Another potential issue is adsorption of the particles at random locations because no particular measures were taken to protect against it. A final possibility is that the assumption that all particles get jammed at the inner edge of the cavity due to electrophoretic forces, as in Figure 1b, is oversimplified. Due to the nanoscopic height of the ring electrodes, the electric field gradients created are much larger than at conventional UMEs. This may lead to additional effects such as dielectric forces, or electroosmosis that can normally be ignored at the salt concentrations employed here. We envision that particles may get trapped on the surface surrounding the cylindrical cavity due to this complex balance of forces. Increasing the height of the top and bottom layers of SiN or employing smaller particles in a device with a thinner Pt layer can diminish this effect. Finally, a true planar ring geometry could be implemented, at the cost of requiring more sophisticated microfabrication equipment.

## Conclusions and Outlook

Here, we explored the use of ring electrodes with a nanoscale width as an alternative tool for detecting insulating particles via current-blockade impact electrochemistry. We argued that the broad distribution of step sizes for a disk can be explained semiquantitatively by a simple model taking into account the non-uniform current distribution over the surface of the electrode together with the resulting non-uniform migrational flux of particles. In order to diminish the edge effect, we designed and fabricated a ring geometry based on a thin strip electrode sandwiched between insulating materials. The current-blockade impact measurements using ring UMEs showed larger and more uniform relative step sizes compared to a disk with the same diameter. In particular, the distribution of step sizes for the ring UME is narrow enough to reliably identify the simultaneous collision of two particles as a double-sized step. Also, the occurrence of a single-step size distribution should allow accurate particle size determination. This geometry further addresses the particle displacement problem that leads to false collision events. The step size uniformity can be further improved by fabricating devices with more optimized geometries. The use of these ring UMEs is not limited to current blockade: the fabrication process is relatively straightforward and can be applied to different materials, such that the advantages of ring UMEs can also be exploited in other impact electrochemistry methods.

## Supporting Information Available

Additional details of the experimental methods and analysis (PDF)
Comsol simulation report (PDF)
