# Supplementary material for: Ring Ultramicroelectrodes for Current-Blockade Particle-Impact Electrochemistry
Source: Anal Chem. 2022 Jul 6;94(28):10168–74. doi: 10.1021/acs.analchem.2c01503 (PMC9310007; doi:10.1021/acs.analchem.2c01503)
Supplement: Supplementary file 1 — ac2c01503_si_001.pdf [file ac2c01503_si_001.pdf]

Supporting Information

Ring ultramicroelectrodes for current-blockade particle-impact electrochemistry

Taghi Moazzenzade, Tieme Walstra, Xiaojun Yang, Jurriaan Huskens* and Serge G. Lemay*

MESA+ Institute and Faculty of Science and Technology, University of Twente, P.O. Box 217, 7500 AE Enschede, The Netherlands

Table of content

1. Detailed fabrication process flow -- p2
2. Measurement setup --- p4
3. Obtaining the step size distribution from eq 4 ------------------------------------- p5
4. Current Step Analysis -- p6
5. Histograms --- p7

1- Detailed fabrication process flow

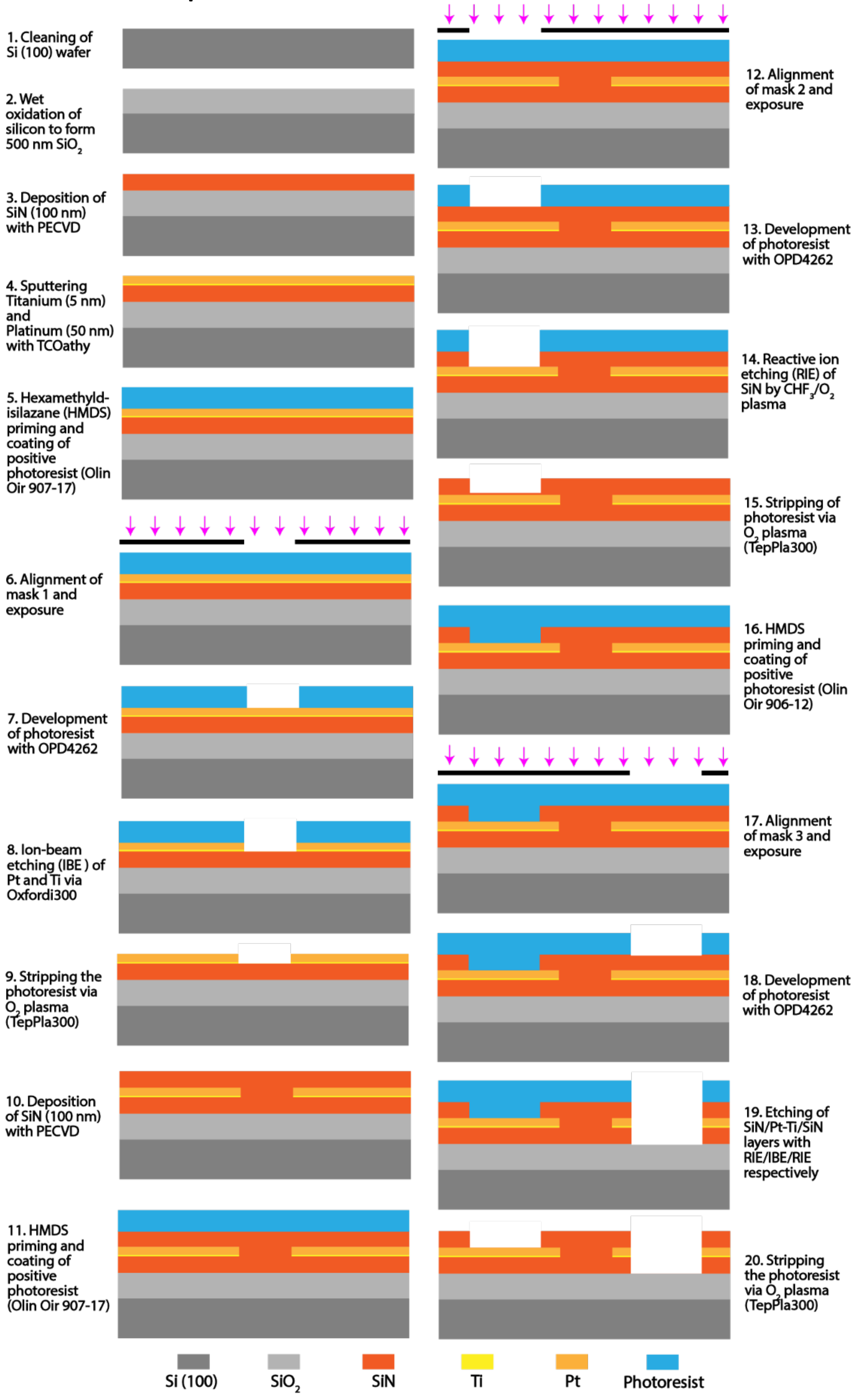

Figure S1. Schematic representation of cross-sections at different steps of the process flow for the fabrication of the disk and ring UMEs; 1- Wafer cleaning, 2- Wet oxidation, 3- SiN deposition, 4- Sputtering of Ti and Pt, 5- Coating of the positive photoresist (Olin Oir 907-17), 6- Alignment and

exposure, 7- Development of the photoresist with OPD4262, 8- Etching Pt and Ti layers *via* IBE, 9- Stripping of the photoresist with O_2 plasma, 10- SiN deposition *via* PECVD, 11- Coating of the photoresist, 12- Alignment and exposure, 13- Development of the photoresist, 14- Etching SiN layer *via* RIE (exposing the disk UMEs and connections), 15- Stripping of the photoresist with O_2 plasma, 16- Coating of the positive photoresist (Olin Oir 906-12), 17- Alignment and exposure, 18- Development of the photoresist, 19- Etching SiN/Pt-Ti/SiN layers using a combination of RIE/IBE/RIE methods (exposing the ring UMEs), 20- Stripping of the photoresist with O_2 plasma.

2- Measurement setup

The chip is placed in a custom stage that includes spring-loaded pins to establish electrical contact to the electrodes. A PDMS cylinder is used as a cell. The pseudo-reference electrode is inserted through the top opening. The contact between the PDMS and the chip surfaces can be checked by optical microscopy to ensure that there is no leakage in the cell.

a

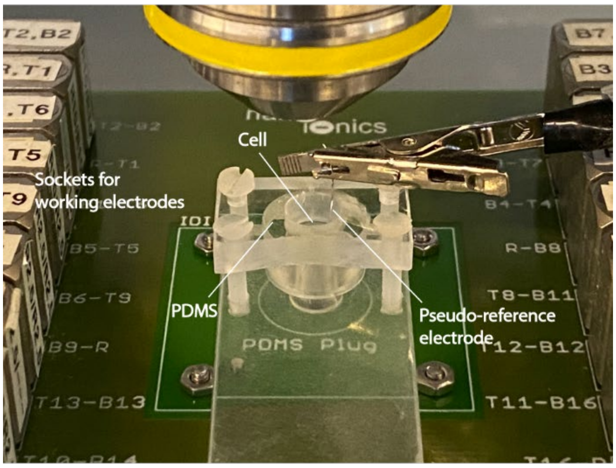

b

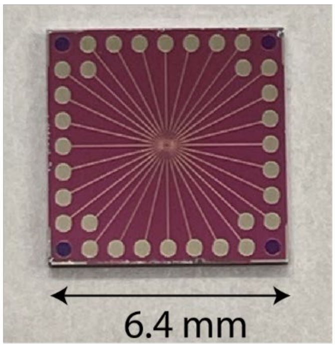

Figure S2. (a) Assembled two-electrode cell. (b) The fabricated device.

3- Obtaining the step size distribution from eq 4

The calculated step size distribution for a disk electrode shown in Figure 3b was obtained from eq 4. Doing so requires knowledge of the quantity $d(\Delta i(r))/dr$, where $\Delta i(r)$ is the current step size for a particle landing at radial coordinate r. In order to determine $\Delta i(r)$, finite-element calculations were performed using COMSOL Multiphysics 5.3a. A report of a typical computation is provided separately. The resulting values are shown as solid symbols in Figure S3. These results were then fitted to a fifth-order polynomial to generate a smooth curve that could be differentiated and input into eq 4. The fitted polynomial had values

$$\Delta i(r) = 0.00140r^5 + 0.00149r^4 - 0.0441r^3 + 0.121r^2 - 0.000896r + 0.614$$

where r has units of micrometers and Δi has units of nA.

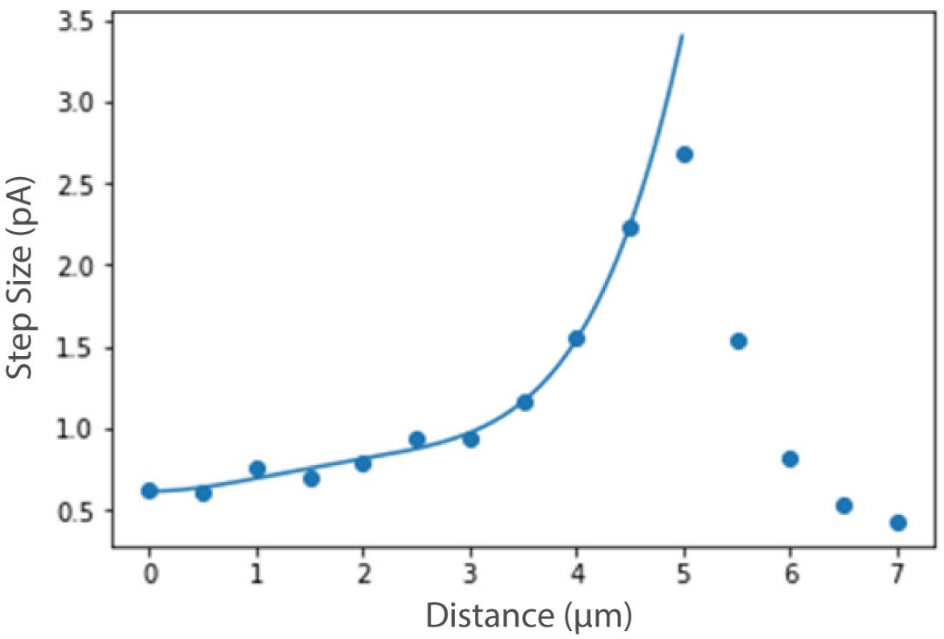

Figure S3: Numerically calculated values of $\Delta i(r)$ and corresponding polynomial fit.

4- Current Step Analysis

The analysis of the chronoamperograms was performed with a self-produced Python program. Generally, the program uses a current-time response as input. It applies a filter to smooth out the noise, differentiates the signal, and identifies the peaks that exceed a certain threshold in the resulting curve. Peaks in this signal correspond to steps in the chronoamperogram.

In the first step, the current-time response is filtered using a Savitzky-Golay filter to remove high-frequency noises and unwanted spikes. The filter fits polynomials through groups of adjacent data points. The order of this polynomial and the window length can be given as parameters as well as the derivative order. In practice, the polynomial order, the window length, and the derivative order were 5, 31, and 1 respectively. Then, the resulting smoothed curves are differentiated and used for step finding (Figure S4).

In the next step, the current step is isolated and an analytical function with a linear baseline is fitted through the data points. This directly gives, among other parameters, the height of the step. The step-like function used for steps analysis is

$$\frac{H}{1+e^{-k(t-t_0)}} + m(t-t_0) + b$$

where t is the independent variable, H is the height of the step, k is the steepness of the step, t_0 is the time at which the step occurs, m is the slope of the baseline current before the step, and b is the baseline value directly after the step. The procedure for analyzing a full chronoamperogram is automated, but the user can also check for false positives caused by noise.

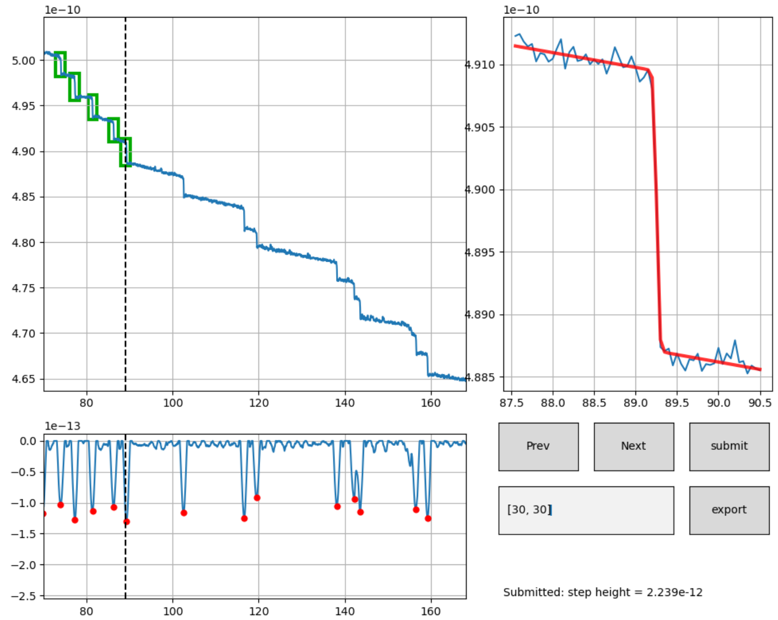

Figure S4: Screenshot of the analysis tool. Top left: raw measurement data (the black dashed line indicates the particular step being analyzed, green boxes indicate previously analyzed steps). Bottom left: derivative of the filtered data (red dots indicate a peak corresponding to a step candidate). Top right: isolated step currently being analyzed (blue) together with its best fit (red). Buttons allow navigating through the complete trace.

5- Histograms

One possible concern is that the magnitude of the events can depend on previous history when multiple particles land in each other's vicinity. However, analyzing lower numbers of steps for each trace results in histograms with a similar shape, indicating that retaining 15 events per trace does not significantly influence the distribution.

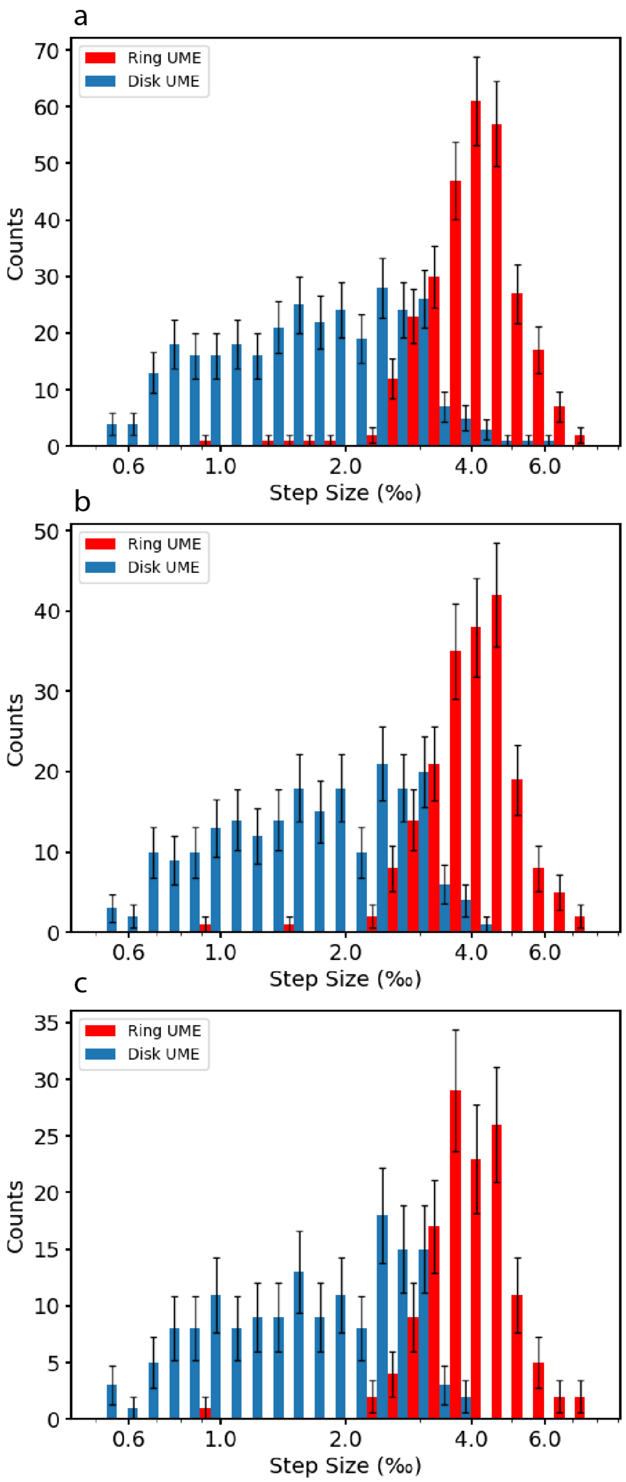

Figure S5. Analysis with a lower number of steps in the measurements of disk and ring UMEs. The histograms shape remains nearly unchanged in both disk and ring UMEs for the first 10 (a), 7 (b), and 5 (c) steps. The x-

axis shows the step size per mille (‰) relative to the baseline current just before the step on a logarithmic scale. The standard deviations of the step size for the first 10, 7, and 5 steps in disk and ring UMEs are (0.24, 0.12), (0.23, 0.12), and (0.23, 0.11), respectively. The smallest steps in the ring measurements can be due to the collision of particles near previously landed particles.

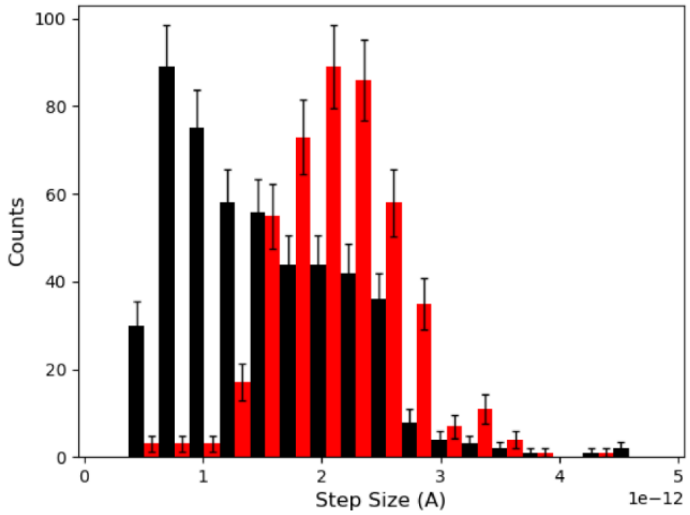

Figure S6. **Histograms of the step sizes for ring and disk UMEs plotted on a linear axis (same data as Figure 3). Note that while the variance in the absolute step size is comparable for both situations, the disk electrode exhibits a broad distribution that varies over a factor of ~5 while the ring electrode exhibits a peaked distribution around 2 pA.**
