# Supplementary material for: Ring Ultramicroelectrodes for Current-Blockade Particle-Impact Electrochemistry
Source: Anal Chem. 2022 Jul 6;94(28):10168–74. doi: 10.1021/acs.analchem.2c01503 (PMC9310007; doi:10.1021/acs.analchem.2c01503)
Supplement: Supplementary file 2 — ac2c01503_si_002.pdf [file ac2c01503_si_002.pdf]

COMSOL MULTIPHYSICS®

Disc UME 1000nm-0-7- report for paper

Report date	Jan 17, 2022 8:29:48 PM

CONTENTS

1. Global Definitions

1.1. Parameters

2. Component 1

2.1. Definitions

2.2. Geometry 1

2.3. Materials

2.4. Transport of Diluted Species

2.5. Mesh 1

3. Study 1

3.1. Stationary

3.2. Solver Configurations

4. Results

4.1. Data Sets

4.2. Derived Values

4.3. Tables

4.4. Plot Groups

1. Global Definitions

Date	Jan 17, 2022 8:02:50 PM

GLOBAL SETTINGS

Name	Disc UME 1000nm-0-7- report for paper.mph
Path	C:\Users\MoazzenzadeT\Desktop\Final final simulation\Disc UME 1000nm-0-7- report for paper.mph
Version	COMSOL Multiphysics 5.5 (Build: 292)

Unit system	SI

USED PRODUCTS

COMSOL Multiphysics

1.1. PARAMETERS

PARAMETERS 1

Name	Expression	Value	Description
D	6.7e-10[m^2/s]	$6.7E-10\ m^2/s$	Diffusion coefficient
F	96485.332[C/mol]	96485 C/mol	Faraday constant
c0	0.67[mol/m^3]	$0.67\ mol/m^3$	Bulk concentration

2. Component 1

Date	Apr 7, 2020 9:40:24 AM

SETTINGS

Description	Value
Unit system	Same as global system
Geometry shape order	Automatic
Avoid inverted elements by curving interior domain elements	Off

SPATIAL FRAME COORDINATES

First	Second	Third
x	y	z

MATERIAL FRAME COORDINATES

First	Second	Third
X	Y	Z

GEOMETRY FRAME COORDINATES

First	Second	Third
Xg	Yg	Zg

MESH FRAME COORDINATES		
**First**	**Second**	**Third**
Xm	Ym	Zm

2.1. DEFINITIONS

2.1.1. Coordinate Systems

Boundary System 1

Coordinate system type	Boundary system
Tag	sys1

COORDINATE NAMES		
**First**	**Second**	**Third**
t1	t2	n

SETTINGS	
**Description**	**Value**
Frame	Geometry configuration

Cylindrical System 2

Coordinate system type	Cylindrical system
Tag	sys2

COORDINATE NAMES		
**First**	**Second**	**Third**
r	phi	a

ORIGIN		
**x (m)**	**y (m)**	**z (m)**
0	0	0

SETTINGS		
**x**	**y**	**z**
0	0	1

SETTINGS		
**x**	**y**	**z**
1	0	0

2.2. GEOMETRY 1

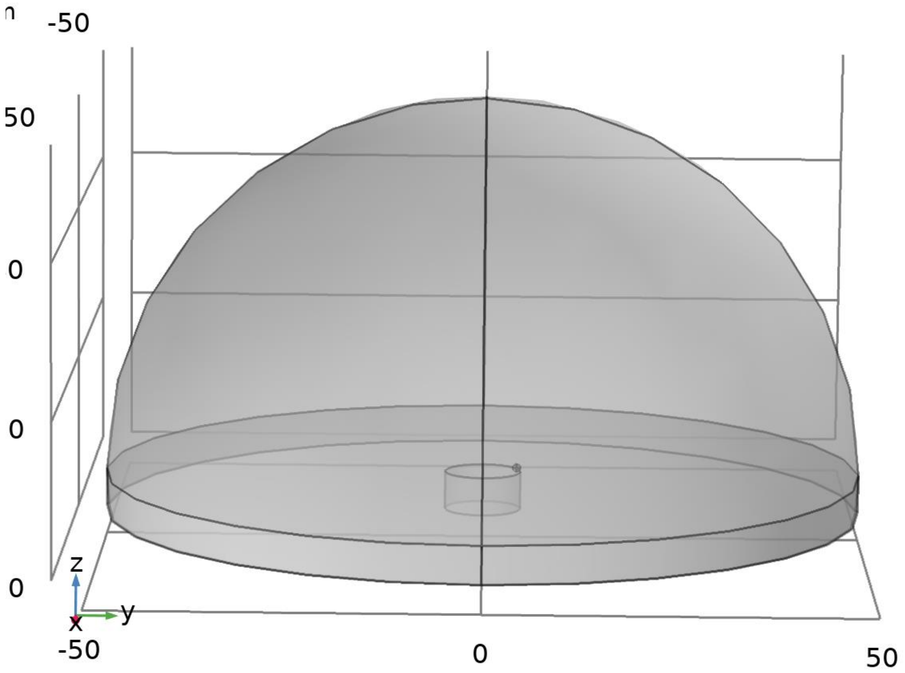

Geometry 1

UNITS

Length unit	µm
Angular unit	deg

GEOMETRY STATISTICS

Description	Value
Space dimension	3
Number of domains	5
Number of boundaries	29
Number of edges	48
Number of vertices	27

2.2.1. Cylinder 1 (cyl1)

POSITION

Description	Value
Position	{0, 0, 0}

AXIS

Description	Value
Axis type	z - axis

SIZE AND SHAPE

Description	Value
Radius	50
Height	5

2.2.2. Sphere 1 (sph1)

POSITION

Description	Value
Position	{0, 0, 5}

AXIS

Description	Value
Axis type	z - axis

SIZE

Description	Value
Radius	50

2.2.3. Partition Objects 1 (par1)

SETTINGS

Description	Value
Keep tool objects	On

2.2.4. Cylinder 2 (cyl2)

POSITION

Description	Value
Position	{0, 0, 0}

AXIS

Description	Value
Axis type	z - axis

SIZE AND SHAPE

Description	Value
Radius	5
Height	5

2.2.5. Cylinder 3 (cyl3)

POSITION

Description	Value
Position	{0, 0, 0}

AXIS

Description	Value
Axis type	z - axis

SIZE AND SHAPE

Description	Value
Radius	5
Height	5

2.2.6. Sphere 2 (sph2)

POSITION

Description	Value
Position	{0, 4.5, 5.52}

AXIS

Description	Value
Axis type	z - axis

SIZE

Description	Value
Radius	0.5

2.3. MATERIALS

2.3.1. H2O (water) [liquid]

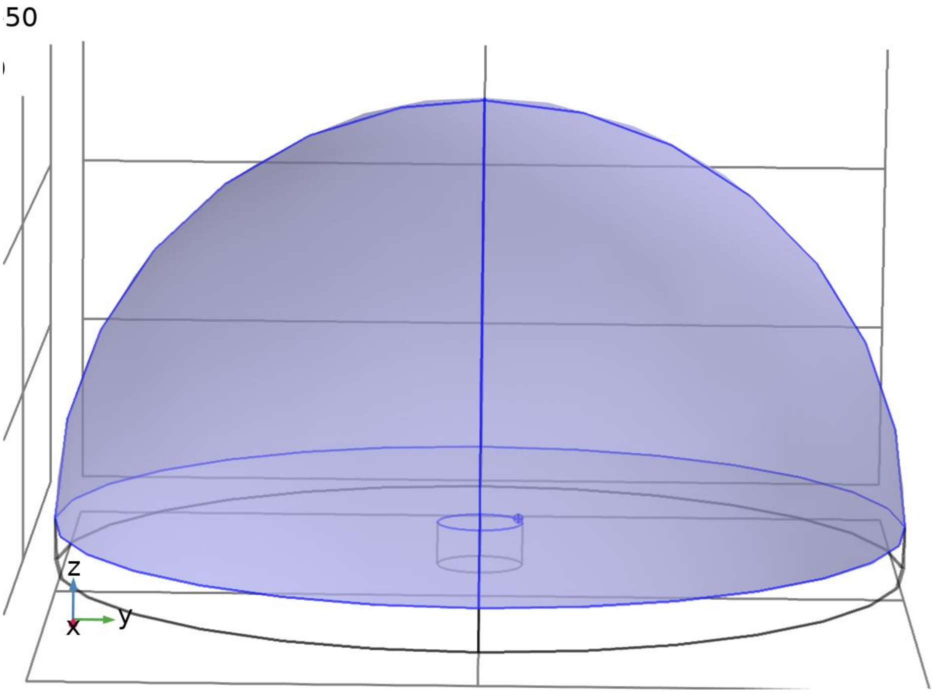

H2O (water) [liquid]

SELECTION

Geometric entity level	Domain
Selection	Geometry geom1: Dimension 3: Domain 3

BASIC SETTINGS

Description	Value
Thermal conductivity	{{k_liquid_2(T[1/K])[W/(m*K)], 0, 0}, {0, k_liquid_2(T[1/K])[W/(m*K)], 0}, {0, 0, k_liquid_2(T[1/K])[W/(m*K)]}}

Coefficient of thermal expansion	{{(alpha_liquid_2(T[1/K])[1/K] + (Tempref - 293[K])*if(abs(T - Tempref)>1e-3, (alpha_liquid_2(T[1/K])[1/K] - alpha_liquid_2(Tempref[1/K])[1/K])/(T - Tempref), d(alpha_liquid_2(T[1/K])[1/K], T)))/(1 + alpha_liquid_2(Tempref[1/K])[1/K]*(Tempref - 293[K])), 0, 0}, {0, (alpha_liquid_2(T[1/K])[1/K] + (Tempref - 293[K])*if(abs(T - Tempref)>1e-3, (alpha_liquid_2(T[1/K])[1/K] - alpha_liquid_2(Tempref[1/K])[1/K])/(T - Tempref), d(alpha_liquid_2(T[1/K])[1/K], T)))/(1 + alpha_liquid_2(Tempref[1/K])[1/K]*(Tempref - 293[K])), 0}, {0, 0, (alpha_liquid_2(T[1/K])[1/K] + (Tempref - 293[K])*if(abs(T - Tempref)>1e-3, (alpha_liquid_2(T[1/K])[1/K] - alpha_liquid_2(Tempref[1/K])[1/K])/(T - Tempref), d(alpha_liquid_2(T[1/K])[1/K], T)))/(1 + alpha_liquid_2(Tempref[1/K])[1/K]*(Tempref - 293[K]))}}
Heat capacity at constant pressure	C_liquid_2(T[1/K])[J/(kg*K)]
HC	HC_liquid_2(T[1/K])[J/(mol*K)]
VP	VP_liquid_2(T[1/K])[Pa]
Density	rho_liquid_2(T[1/K])[kg/m^3]
TD	TD_liquid_2(T[1/K])[m^2/s]
Dynamic viscosity	eta_liquid_1(T[1/K])[Pa*s]

FUNCTIONS

Function name	Type
k_liquid_2	Piecewise
alpha_liquid_2	Piecewise
C_liquid_2	Piecewise
HC_liquid_2	Piecewise
VP_liquid_2	Piecewise
rho_liquid_2	Piecewise
TD_liquid_2	Piecewise
eta_liquid_1	Piecewise

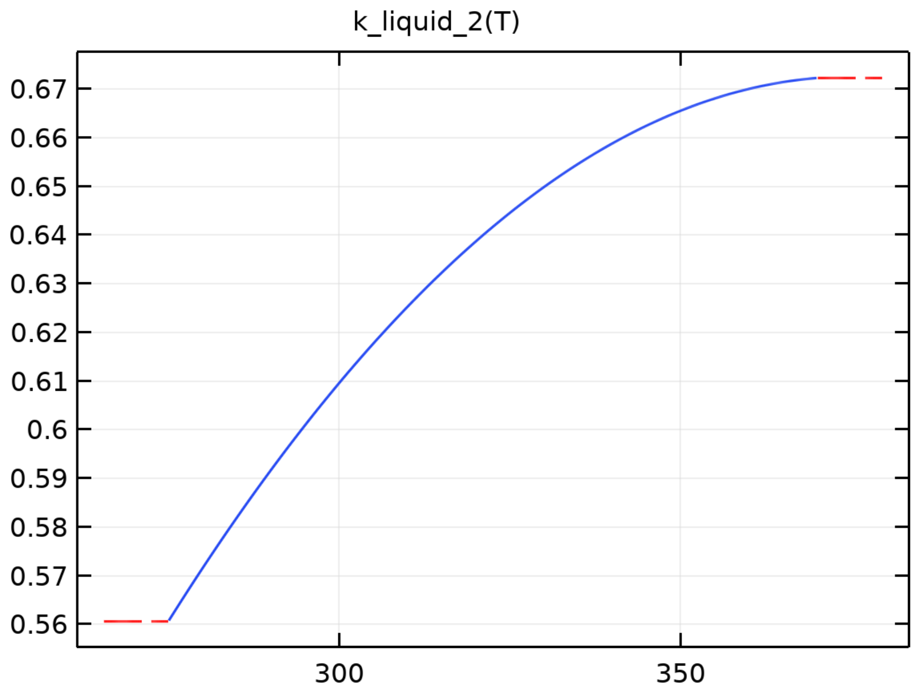

k_liquid_2

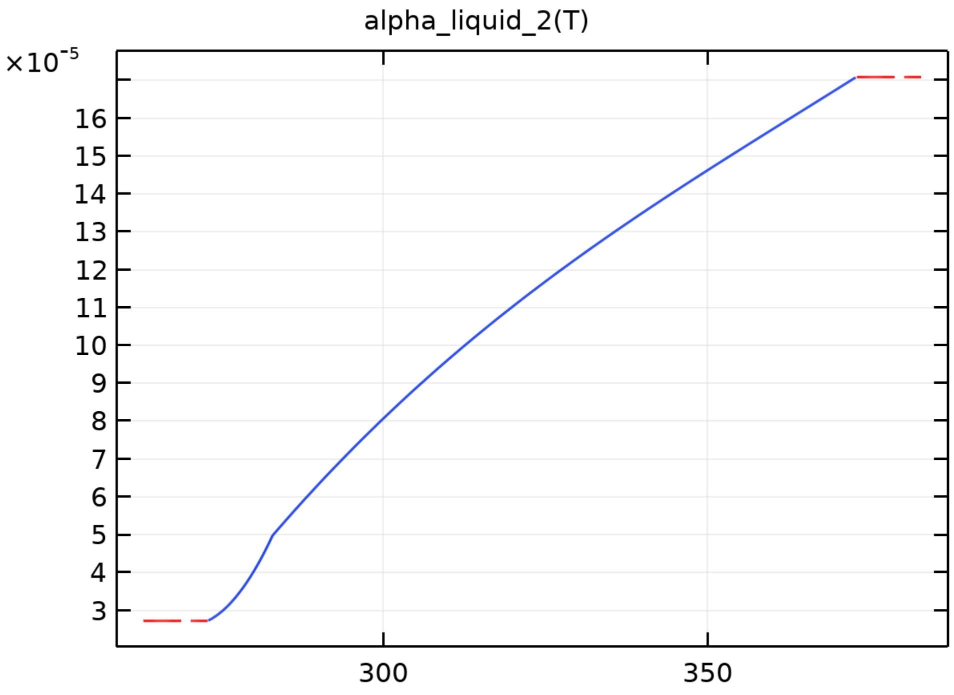

alpha_liquid_2

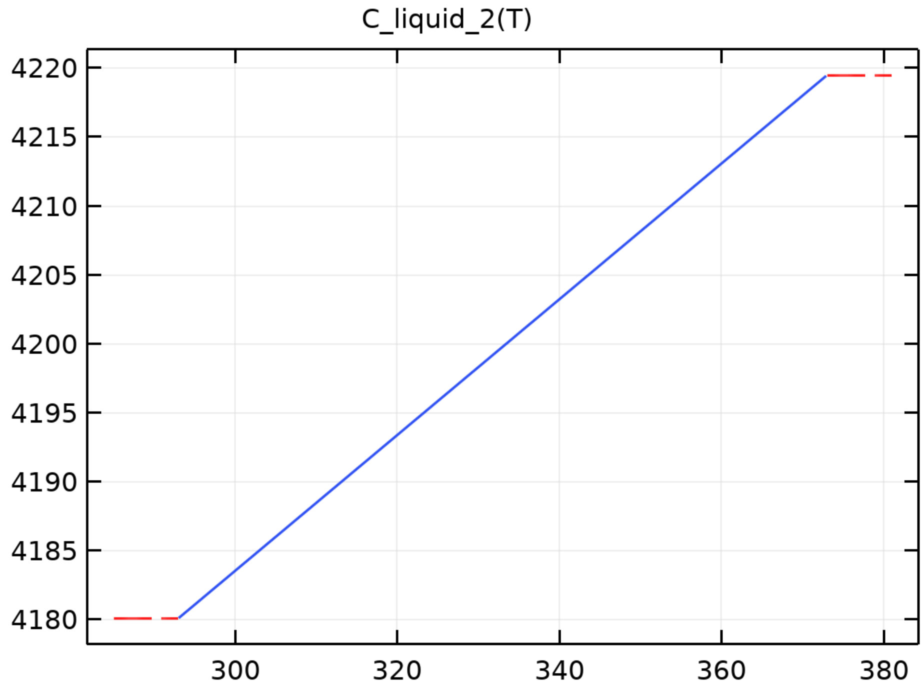

C_liquid_2

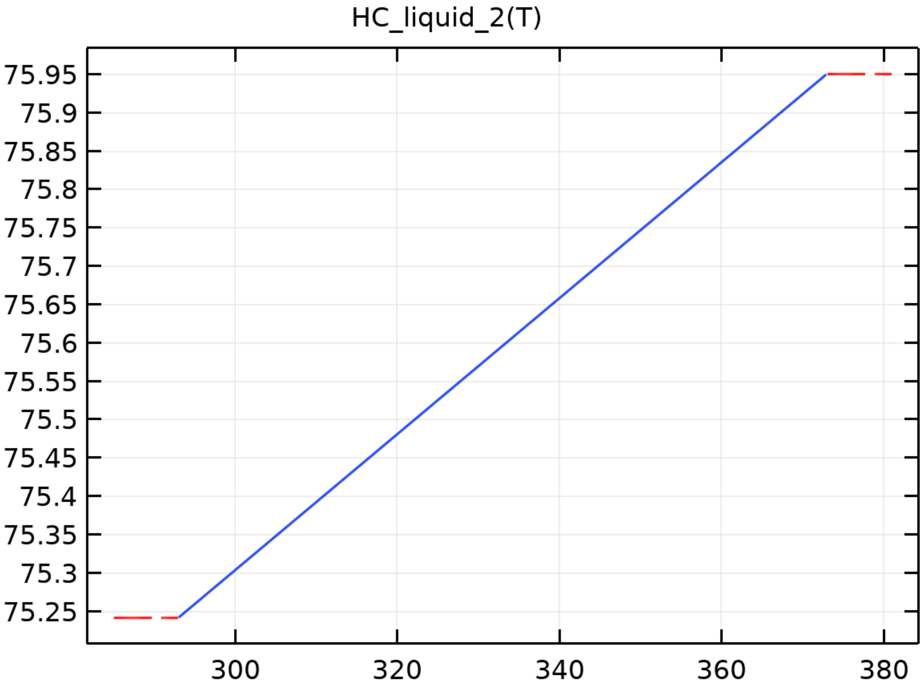

HC_liquid_2

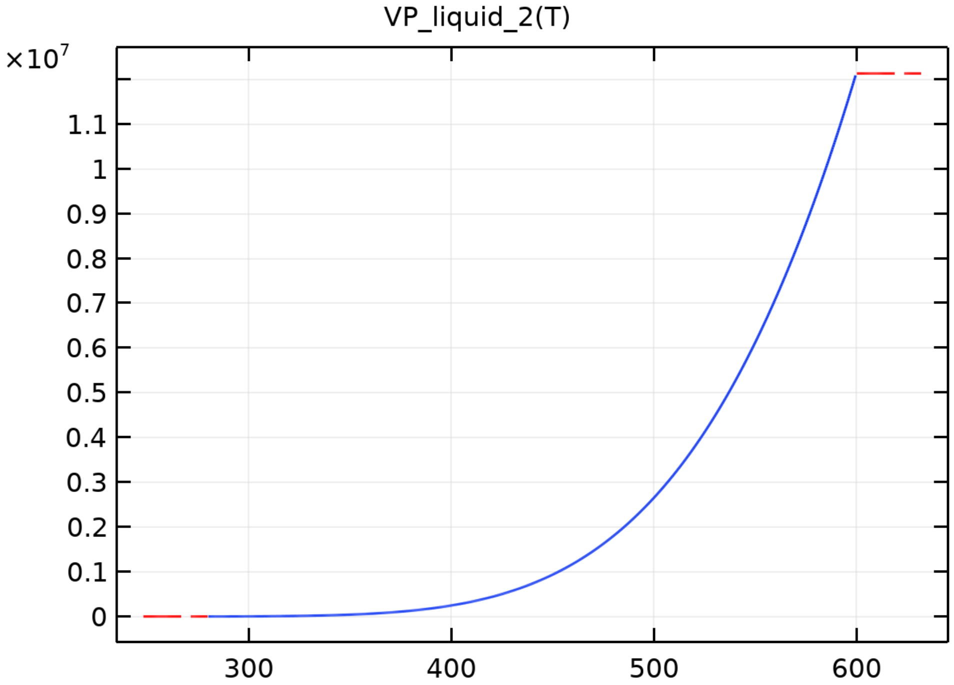

VP_liquid_2

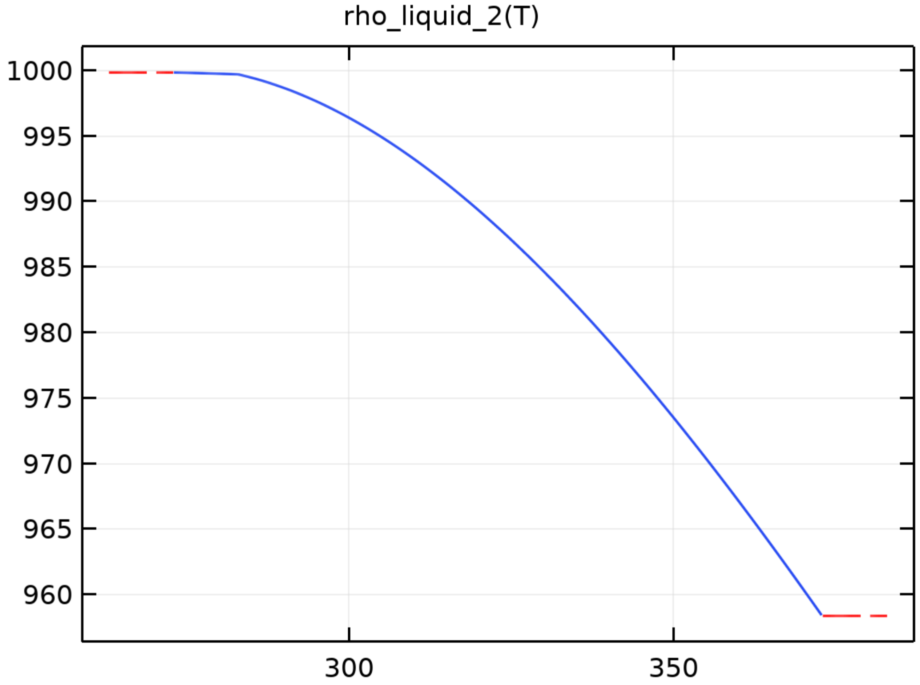

rho_liquid_2

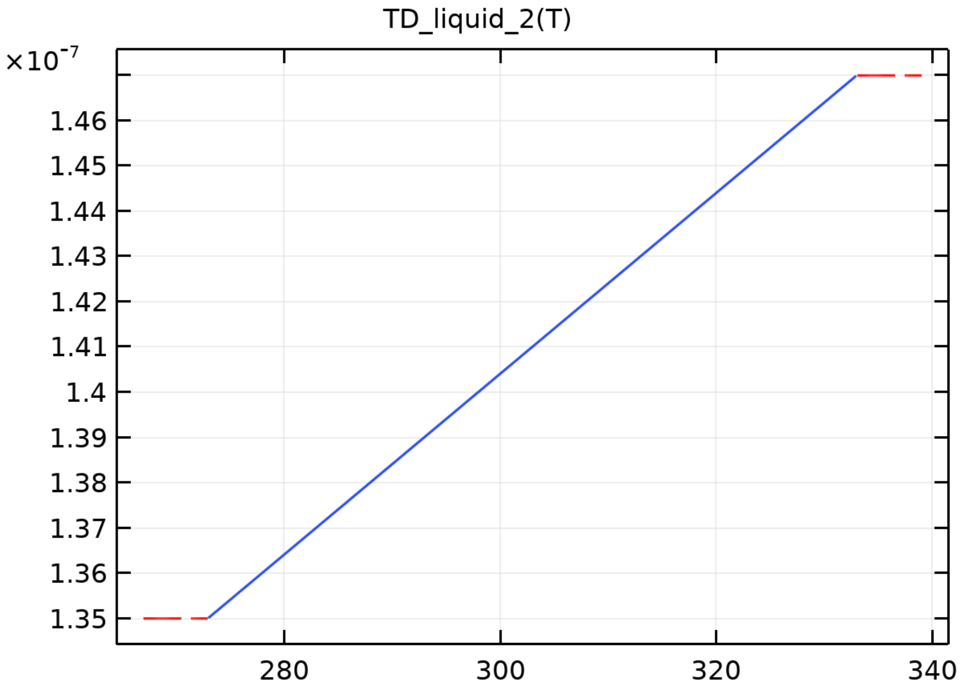

TD_liquid_2

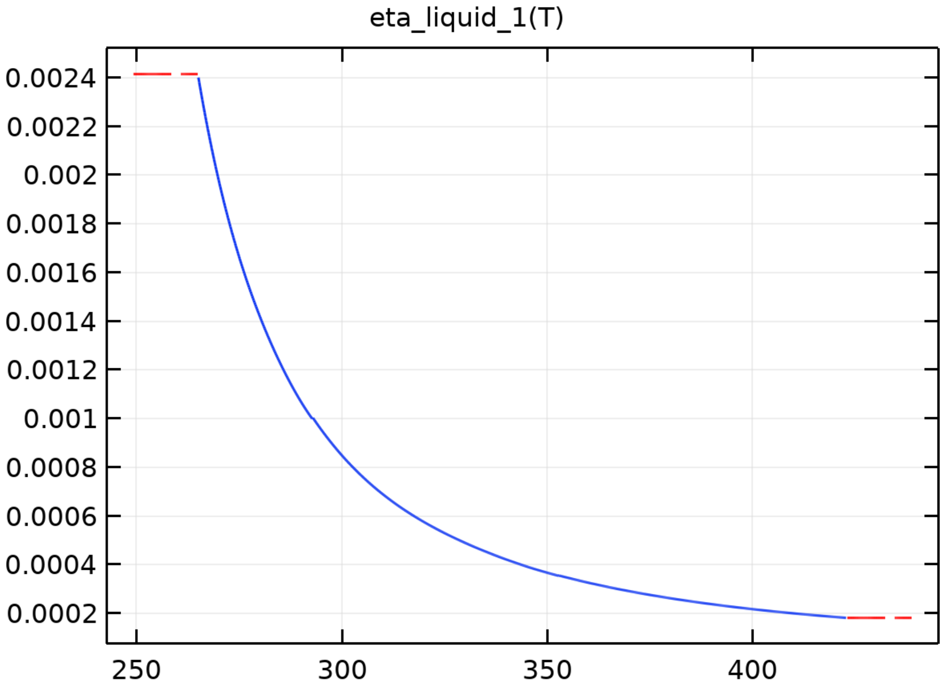

eta_liquid_1

THERMAL EXPANSION SETTINGS

Description	Value
Tangent coefficient of thermal expansion	{{CTE_liquid_2(T[1/K])[1/K], 0, 0}, {0, CTE_liquid_2(T[1/K])[1/K], 0}, {0, 0, CTE_liquid_2(T[1/K])[1/K]}}

Thermal strain	{{(dL_liquid_2(T[1/K]) - dL_liquid_2(Tempref[1/K]))/(1 + dL_liquid_2(Tempref[1/K])), 0, 0}, {0, (dL_liquid_2(T[1/K]) - dL_liquid_2(Tempref[1/K]))/(1 + dL_liquid_2(Tempref[1/K])), 0}, {0, 0, (dL_liquid_2(T[1/K]) - dL_liquid_2(Tempref[1/K]))/(1 + dL_liquid_2(Tempref[1/K]))}}
Isotropic tangent coefficient of thermal expansion	CTE_liquid_2(T)
Isotropic thermal strain	(dL_liquid_2(T) - dL_liquid_2(Tempref))/(1 + dL_liquid_2(Tempref))

FUNCTIONS

Function name	Type
dL_liquid_2	Piecewise
CTE_liquid_2	Piecewise

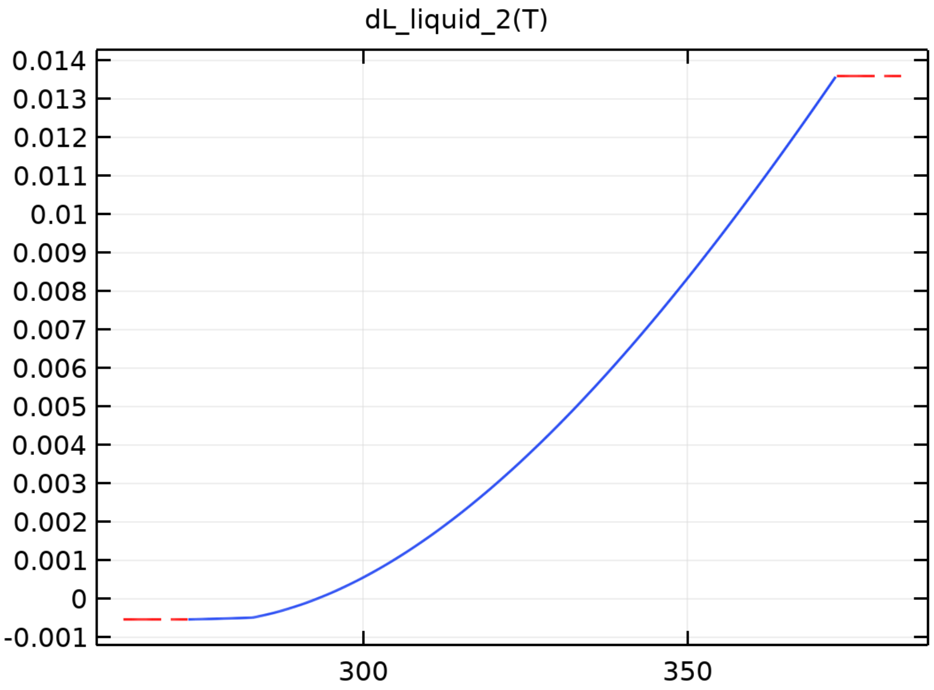

dL_liquid_2

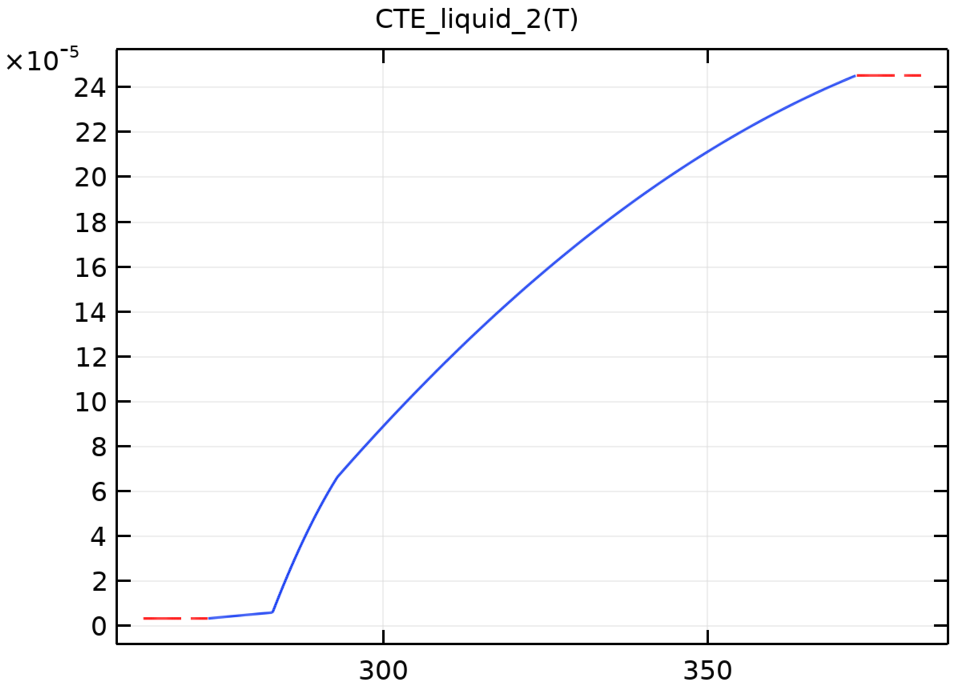

CTE_liquid_2

2.3.2. Pt - Platinum

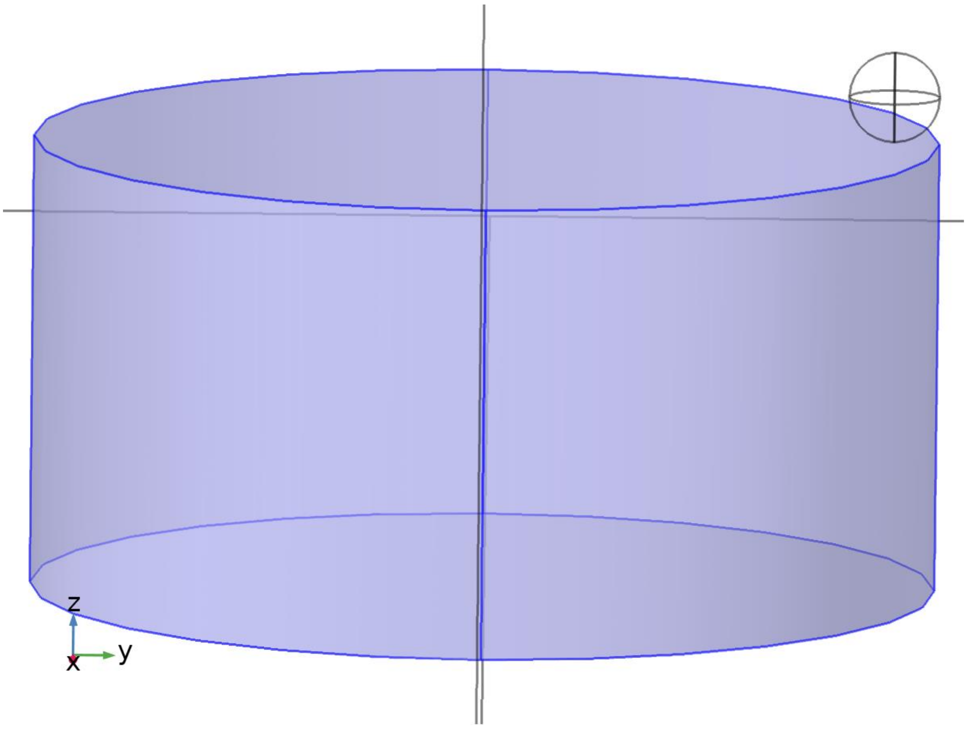

Pt - Platinum

SELECTION

Geometric entity level	Domain
Selection	Geometry geom1: Dimension 3: Domain 4

BASIC SETTINGS

Description	Value
Electrical conductivity	{{8.9e6[S/m], 0, 0}, {0, 8.9e6[S/m], 0}, {0, 0, 8.9e6[S/m]}}
Coefficient of thermal expansion	{{8.80e-6[1/K], 0, 0}, {0, 8.80e-6[1/K], 0}, {0, 0, 8.80e-6[1/K]}}
Heat capacity at constant pressure	133[J/(kg*K)]
Density	21450[kg/m^3]
Thermal conductivity	{{71.6[W/(m*K)], 0, 0}, {0, 71.6[W/(m*K)], 0}, {0, 0, 71.6[W/(m*K)]}}

YOUNG'S MODULUS AND POISSON'S RATIO SETTINGS

Description	Value
Young's modulus	168e9[Pa]
Poisson's ratio	0.38

2.3.3. SiO2 - Silicon oxide

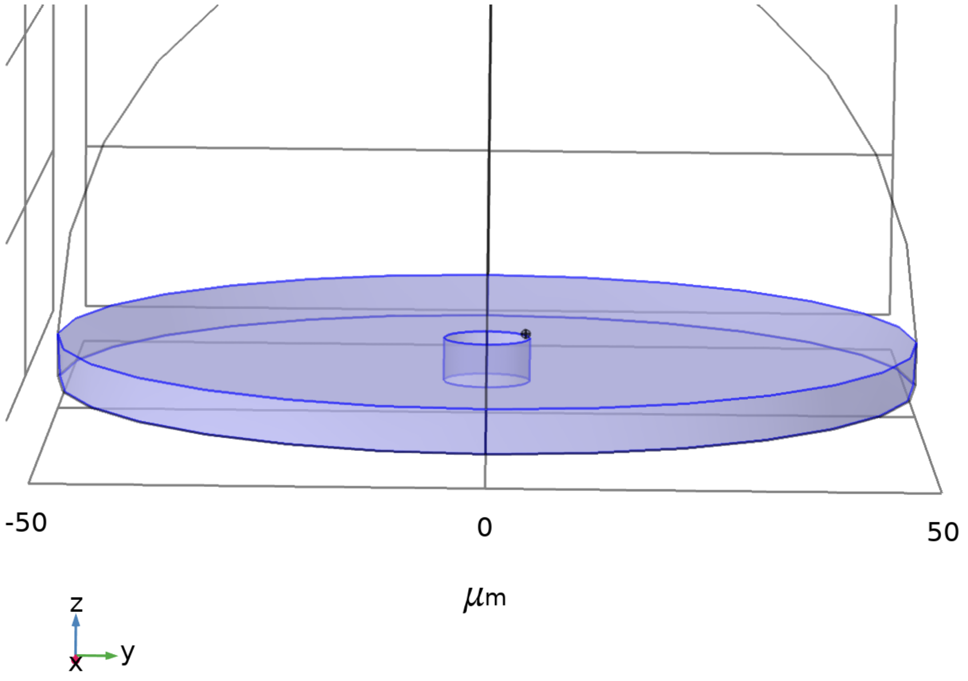

SiO2 - Silicon oxide

SELECTION

Geometric entity level	Domain

Selection	Geometry geom1: Dimension 3: Domain 2

BASIC SETTINGS

Description	Value
Electrical conductivity	{{0[S/m], 0, 0}, {0, 0[S/m], 0}, {0, 0, 0[S/m]}}
Coefficient of thermal expansion	{{0.5e-6[1/K], 0, 0}, {0, 0.5e-6[1/K], 0}, {0, 0, 0.5e-6[1/K]}}
Heat capacity at constant pressure	730[J/(kg*K)]
Relative permittivity	{{4.2, 0, 0}, {0, 4.2, 0}, {0, 0, 4.2}}
Density	2200[kg/m^3]
Thermal conductivity	{{1.4[W/(m*K)], 0, 0}, {0, 1.4[W/(m*K)], 0}, {0, 0, 1.4[W/(m*K)]}}

YOUNG'S MODULUS AND POISSON'S RATIO SETTINGS

Description	Value
Young's modulus	70e9[Pa]
Poisson's ratio	0.17

2.3.4. Polystyrene (PS) [solid]

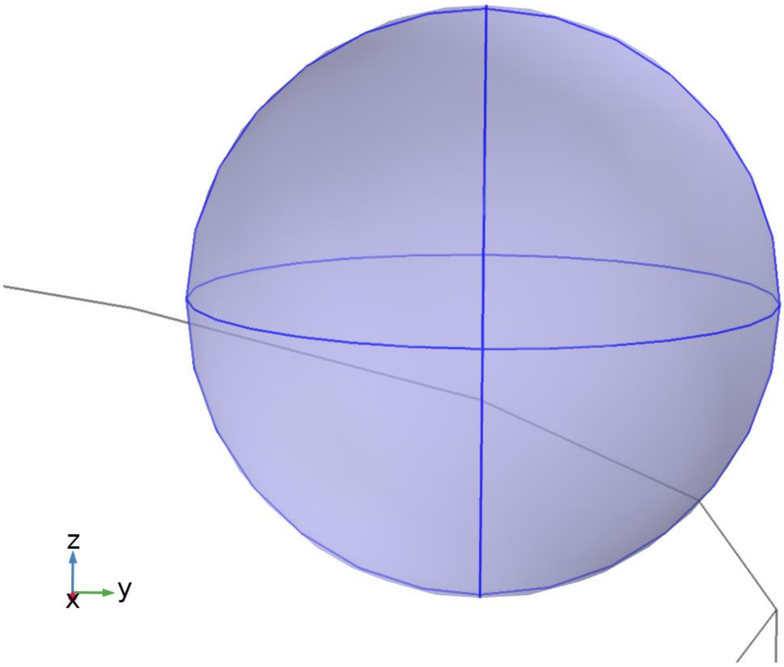

Polystyrene (PS) [solid]

SELECTION

Geometric entity level	Domain
Selection	Geometry geom1: Dimension 3: Domain 5

BASIC SETTINGS

Description	Value
TD	TD(T[1/K])[m^2/s]

FUNCTIONS

Function name	Type
TD	Piecewise

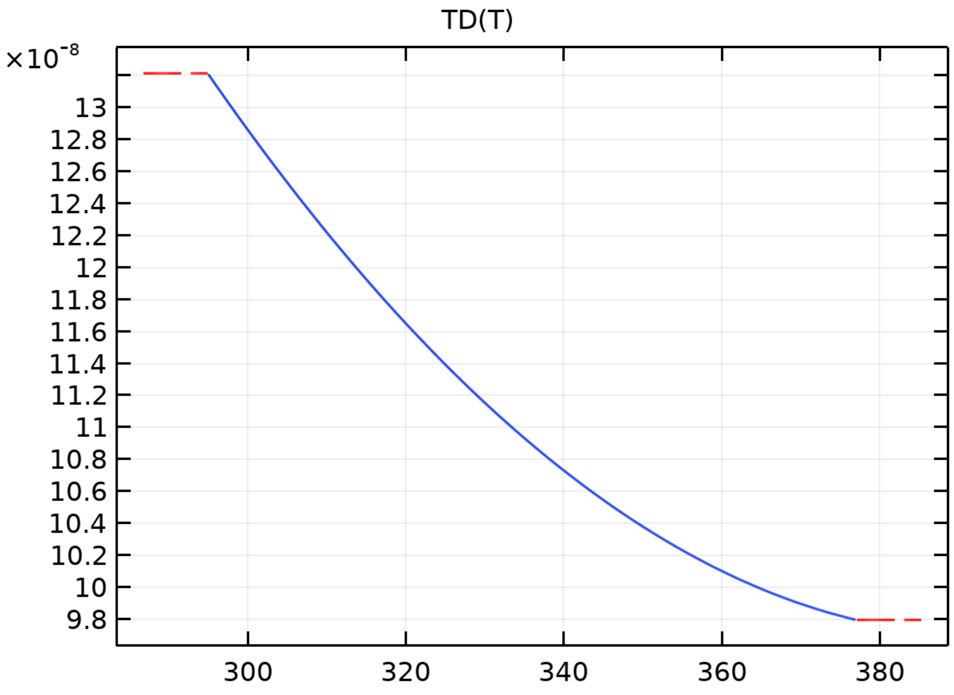

TD

YOUNG'S MODULUS AND POISSON'S RATIO SETTINGS

Description	Value
Young's modulus	E(T[1/K])[Pa]
Poisson's ratio	nu(T[1/K])

FUNCTIONS

Function name	Type
E	Piecewise
nu	Piecewise

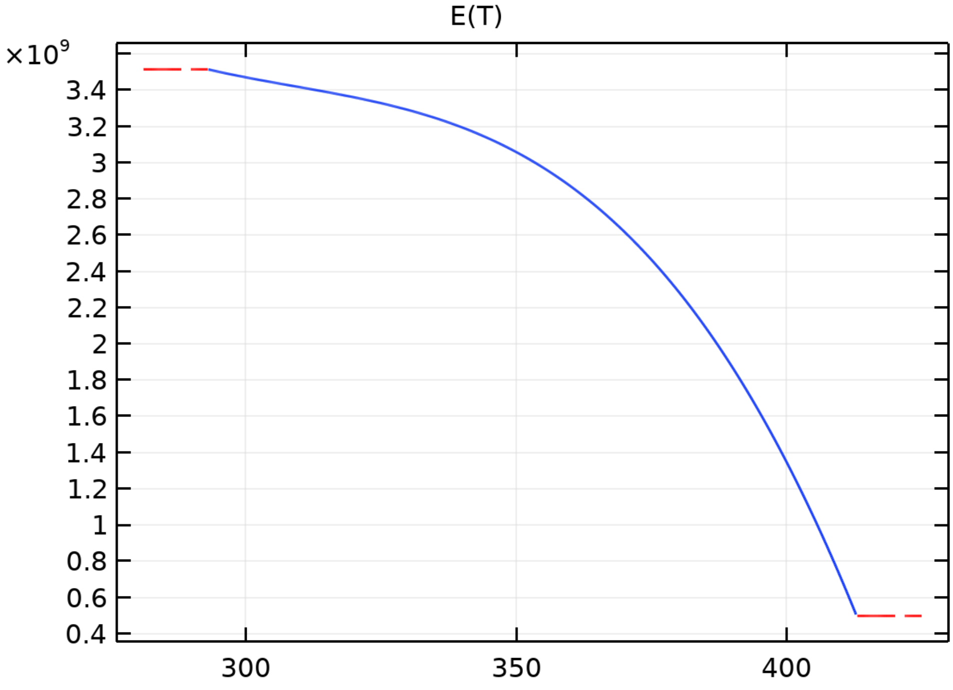

E

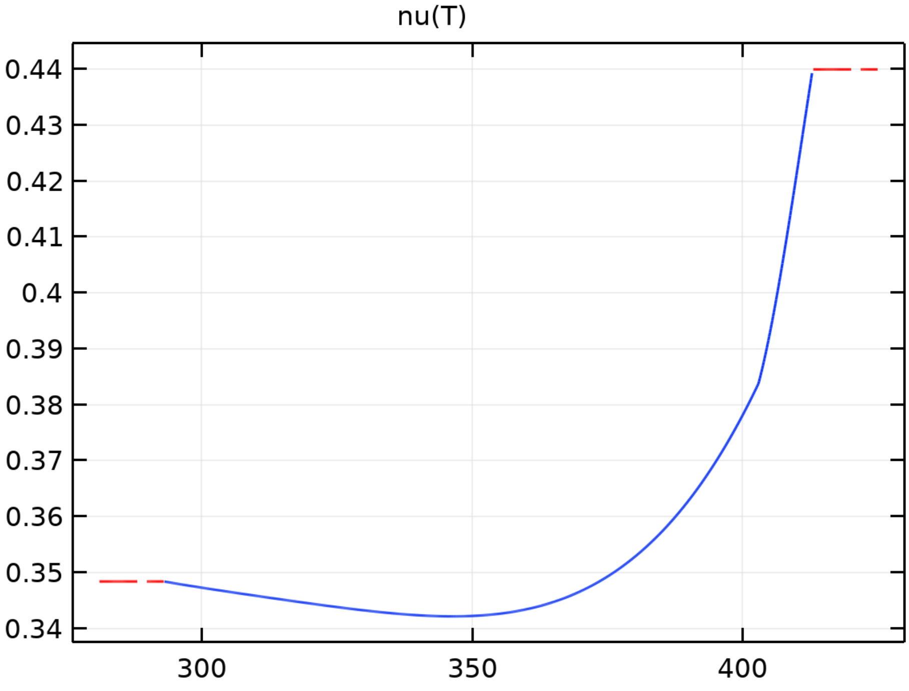

nu

BULK MODULUS AND SHEAR MODULUS SETTINGS

Description	Value
Bulk modulus	kappa(T[1/K])[Pa]
Shear modulus	mu(T[1/K])[Pa]

FUNCTIONS

Function name	Type
mu	Piecewise
kappa	Piecewise

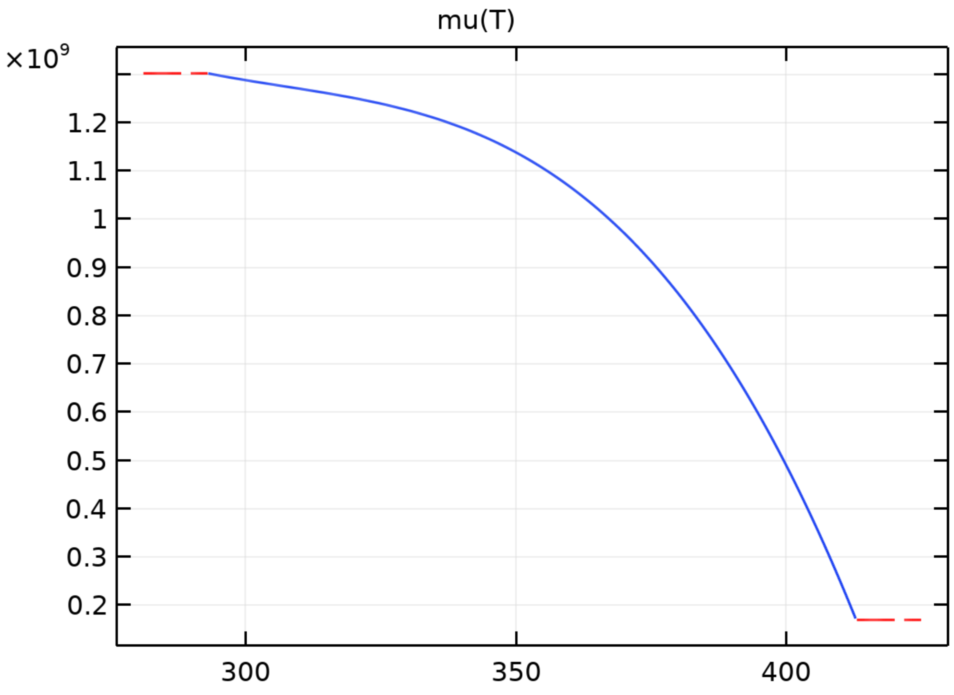

mu

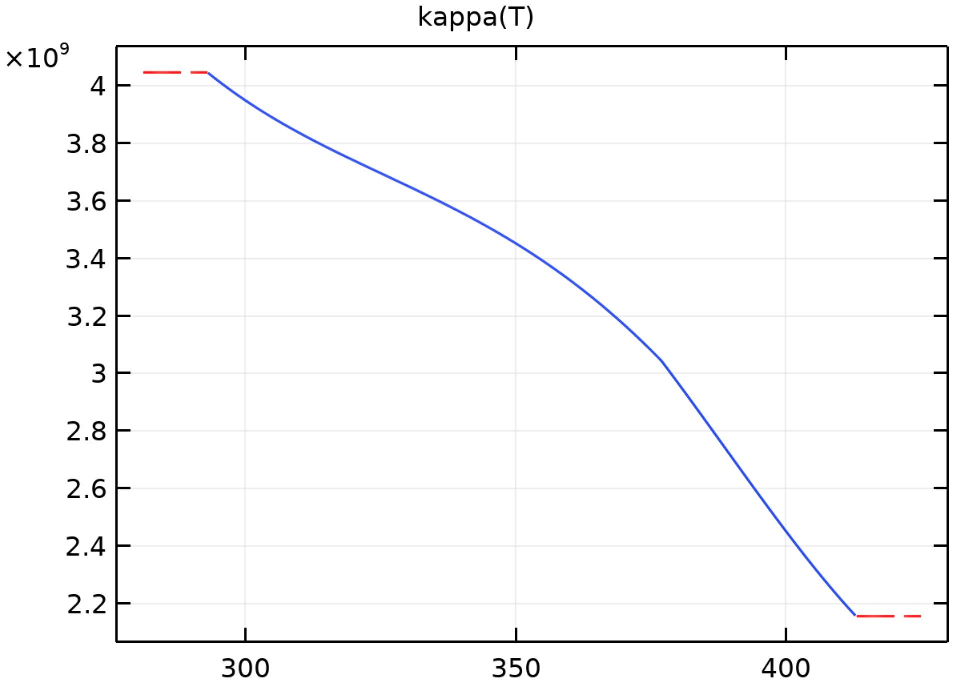

kappa

2.4. TRANSPORT OF DILUTED SPECIES

USED PRODUCTS

COMSOL Multiphysics

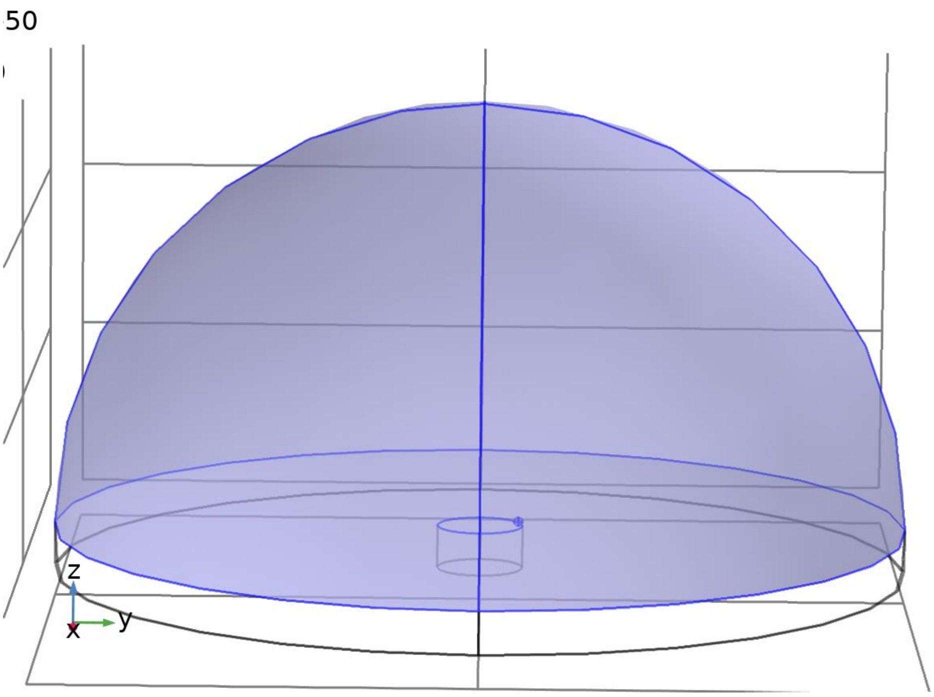

Transport of Diluted Species

SELECTION

Geometric entity level	Domain
Selection	Geometry geom1: Dimension 3: Domain 3

EQUATIONS

$$\nabla \cdot \mathbf{J}_i + \mathbf{u} \cdot \nabla c_i = R_i$$

$$\mathbf{J}_i = -D_i \nabla c_i$$

2.4.1. Interface settings

Discretization

SETTINGS

Description	Value
Concentration	Linear

Transport mechanisms

SETTINGS

Description	Value
Convection	On
Migration in electric field	Off
Mass transfer in porous media	Off

2.4.2. Variables

Name	Expression	Unit	Description	Selection	Details
tds.R_c	0	mol/(m³·s)	Total rate expression	Domain 3	+ operation
tds.cP_c	0	mol/kg	Concentration species absorbed to the solid	Domain 3	+ operation
tds.cP_c	0	mol/kg	Concentration species absorbed to the solid	Boundaries 6–8, 13–17, 20, 22–26	+ operation
tds.KP_c	0	m³/kg	Adsorption isotherm, first concentration derivative	Domain 3	+ operation

tds.KP_c	0	m^3/kg	Adsorption isotherm, first concentration derivative	Boundaries 6–8, 13–17, 20, 22–26	+ operation
tds.epsilon_p	1	1	Porosity	Domain 3	
tds.theta	tds.epsilon_p	1	Liquid volume fraction	Domain 3	
tds.av	0	1	Gas volume fraction	Domain 3	
tds.nx	unx	1	Normal vector, x component	Boundaries 7, 13–17, 23–26	
tds.ny	uny	1	Normal vector, y component	Boundaries 7, 13–17, 23–26	
tds.nz	unz	1	Normal vector, z component	Boundaries 7, 13–17, 23–26	
tds.nx	dnx	1	Normal vector, x component	Boundaries 6, 8, 20, 22	
tds.ny	dny	1	Normal vector, y component	Boundaries 6, 8, 20, 22	
tds.nz	dnz	1	Normal vector, z component	Boundaries 6, 8, 20, 22	
tds.nxmesh	unxmesh	1	Normal vector (mesh), x component	Boundaries 7, 13–17, 23–26	
tds.nymesh	unymesh	1	Normal vector (mesh), y component	Boundaries 7, 13–17, 23–26	
tds.nzmesh	unzmesh	1	Normal vector (mesh), z component	Boundaries 7, 13–17, 23–26	
tds.nxmesh	dnxmesh	1	Normal vector (mesh), x component	Boundaries 6, 8, 20, 22	
tds.nymesh	dnymesh	1	Normal vector (mesh), y component	Boundaries 6, 8, 20, 22	

tds.nzmesh	dnzmesh	1	Normal vector (mesh), z component	Boundaries 6, 8, 20, 22	
tds.nxc	-root.nxc/tds.ncLen	1	Normal vector, x component	Boundaries 7, 13–17, 23–26	
tds.nyc	-root.nyc/tds.ncLen	1	Normal vector, y component	Boundaries 7, 13–17, 23–26	
tds.nzc	-root.nzc/tds.ncLen	1	Normal vector, z component	Boundaries 7, 13–17, 23–26	
tds.nxc	root.nxc/tds.ncLen	1	Normal vector, x component	Boundaries 6, 8, 20, 22	
tds.nyc	root.nyc/tds.ncLen	1	Normal vector, y component	Boundaries 6, 8, 20, 22	
tds.nzc	root.nzc/tds.ncLen	1	Normal vector, z component	Boundaries 6, 8, 20, 22	
tds.ncLen	sqrt(root.nxc^2+root.nyc^2+root.nzc^2+eps)	1	Help variable	Boundaries 6–8, 13–17, 20, 22–26	
tds.cbf_c	0	mol/(m²·s)	Convective boundary flux	Boundaries 6–8, 13–17, 20, 22–26	
tds.u	0	m/s	Velocity field, x component	Domain 3	
tds.v	0	m/s	Velocity field, y component	Domain 3	
tds.w	0	m/s	Velocity field, z component	Domain 3	

2.4.3. Transport Properties 1

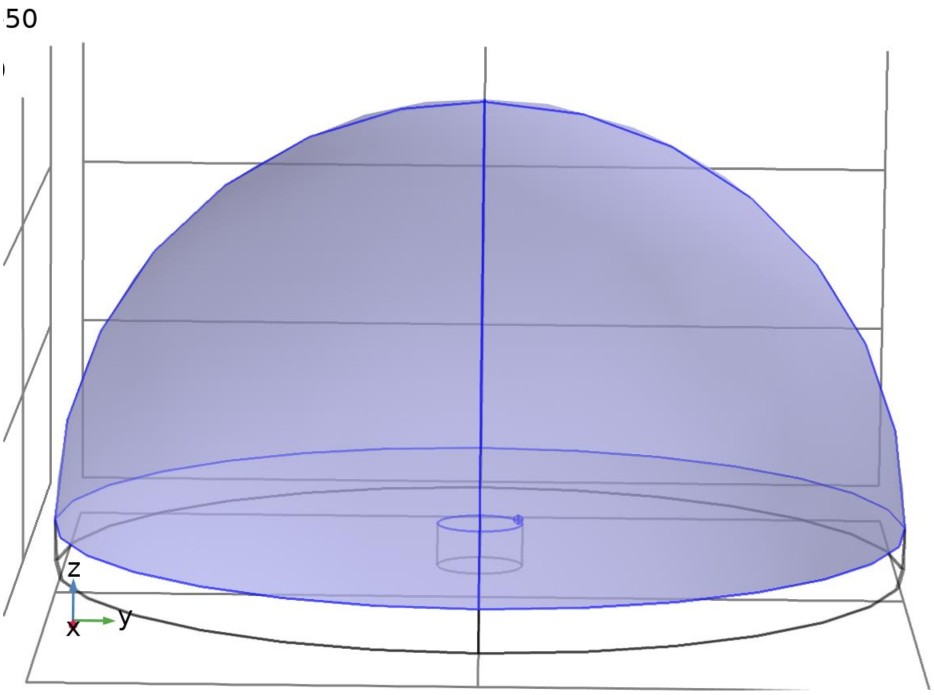

Transport Properties 1

SELECTION

Geometric entity level	Domain
Selection	Geometry geom1: Dimension 3: All domains

EQUATIONS

$$\nabla \cdot \mathbf{J}_i + \mathbf{u} \cdot \nabla c_i = R_i$$

$$\mathbf{J}_i = -D_i \nabla c_i$$

Convection

SETTINGS

**Description**	**Value**
Velocity field	User defined
Velocity field	{0, 0, 0}

Diffusion

SETTINGS

**Description**	**Value**
Material	H2O (water) [liquid] (mat1)
Diffusion coefficient	User defined

Diffusion coefficient	{{D, 0, 0}, {0, D, 0}, {0, 0, D}}

Coordinate system selection

SETTINGS

Description	Value
Coordinate system	Global coordinate system

Model input

SETTINGS

Description	Value
Temperature	Common model input

Variables

Name	Expression	Unit	Description	Selection	Details
domflux.cx	tds.dflux_cx	mol/(m²·s)	Domain flux, x component	Domain 3	
domflux.cy	tds.dflux_cy	mol/(m²·s)	Domain flux, y component	Domain 3	
domflux.cz	tds.dflux_cz	mol/(m²·s)	Domain flux, z component	Domain 3	
tds.ndflux_c	tds.bndFlux_c	mol/(m²·s)	Normal diffusive flux	Boundaries 6–8, 13–17, 20, 22–26	
tds.ncflux_c	tds.cflux_cx*tds.nxc+tds.cflux_cy*tds.nyc+tds.cflux_cz*tds.nzc	mol/(m²·s)	Normal convective flux	Boundaries 6–8, 13–17, 20, 22–26	
tds.ntflux_c	tds.bndFlux_c+tds.cflux_cx*tds.nxc+tds.cflux_cy*tds.nyc+tds.cflux_cz*tds.nzc	mol/(m²·s)	Normal total flux	Boundaries 6–8, 13–17, 20, 22–26	

tds.u	model.input.u1	m/s	Velocity field, x component	Domain 3	Meta
tds.v	model.input.u2	m/s	Velocity field, y component	Domain 3	Meta
tds.w	model.input.u3	m/s	Velocity field, z component	Domain 3	Meta
tds.bndFlux_c	-dflux_spatial(c)	mol/(m²·s)	Boundary flux	Boundaries 6, 8, 20, 22	
tds.bndFlux_c	-uflux_spatial(c)	mol/(m²·s)	Boundary flux	Boundaries 7, 13–17, 23–26	
tds.D_cxx	D	m²/s	Diffusion coefficient, xx component	Domain 3	+ operation
tds.D_cyx	0	m²/s	Diffusion coefficient, yx component	Domain 3	+ operation
tds.D_czx	0	m²/s	Diffusion coefficient, zx component	Domain 3	+ operation
tds.D_cxy	0	m²/s	Diffusion coefficient, xy component	Domain 3	+ operation
tds.D_cyy	D	m²/s	Diffusion coefficient, yy component	Domain 3	+ operation
tds.D_czy	0	m²/s	Diffusion coefficient, zy	Domain 3	+ operation

			component		
tds.D_cxz	0	m²/s	Diffusion coefficient, xz component	Domain 3	+ operation
tds.D_cyz	0	m²/s	Diffusion coefficient, yz component	Domain 3	+ operation
tds.D_czz	D	m²/s	Diffusion coefficient, zz component	Domain 3	+ operation
tds.Dav_c	(tds.D_cxx+tds.D_cyy+tds.D_czz)/3	m²/s	Average diffusion coefficient	Domain 3	
tds.tflux_cx	tds.dflux_cx+tds.cflux_cx	mol/(m²·s)	Total flux, x component	Domain 3	+ operation
tds.tflux_cy	tds.dflux_cy+tds.cflux_cy	mol/(m²·s)	Total flux, y component	Domain 3	+ operation
tds.tflux_cz	tds.dflux_cz+tds.cflux_cz	mol/(m²·s)	Total flux, z component	Domain 3	+ operation
tds.dfluxMag_c	sqrt(tds.dflux_cx^2+tds.dflux_cy^2+tds.dflux_cz^2)	mol/(m²·s)	Diffusive flux magnitude	Domain 3	
tds.tfluxMag_c	sqrt(tds.tflux_cx^2+tds.tflux_cy^2+tds.tflux_cz^2)	mol/(m²·s)	Total flux magnitude	Domain 3	
tds.dflux_cx	-tds.D_cxx*cx-tds.D_cxy*cy-tds.D_cxz*cz	mol/(m²·s)	Diffusive flux, x component	Domain 3	
tds.dflux_cy	-tds.D_cyx*cx-tds.D_cyy*cy-tds.D_cyz*cz	mol/(m²·s)	Diffusive flux, y component	Domain 3	

tds.dflux_cz	-tds.D_czx*cx-tds.D_czy*cy-tds.D_czz*cz	mol/(m²·s)	Diffusive flux, z component	Domain 3	
tds.grad_cx	cx	mol/m⁴	Concentration gradient, x component	Domain 3	
tds.grad_cy	cy	mol/m⁴	Concentration gradient, y component	Domain 3	
tds.grad_cz	cz	mol/m⁴	Concentration gradient, z component	Domain 3	
tds.cflux_cx	c*tds.u	mol/(m²·s)	Convective flux, x component	Domain 3	
tds.cflux_cy	c*tds.v	mol/(m²·s)	Convective flux, y component	Domain 3	
tds.cflux_cz	c*tds.w	mol/(m²·s)	Convective flux, z component	Domain 3	
tds.cfluxMag_c	sqrt(tds.cflux_cx^2+tds.cflux_cy^2+tds.cflux_cz^2)	mol/(m²·s)	Convective flux magnitude	Domain 3	
tds.Res_c	tds.u*cx+tds.v*cy+tds.w*cz-tds.R_c	mol/(m³·s)	Equation residual	Domain 3	

Shape functions

Name	Shape function	Unit	Description	Shape frame	Selection
c	Lagrange (Linear)	mol/m³	Concentration	Spatial	Domain 3

Weak expressions

Weak expression	Integration order	Integration frame	Selection

tds.dflux_cx*test(cx)+tds.dflux_cy*test(cy)+tds.dflux_cz*test(cz)	2	Spatial	Domain 3
-<br>(tds.u*cx+tds.v*cy+tds.w*cz)*test(c)*(isScalingSystemDomain==0)	2	Spatial	Domain 3
tds.cbf_c*test(c)	2	Spatial	Boundaries 6–8, 13–17, 20, 22–26
tds.streamline*(isScalingSystemDomain==0)	2	Spatial	Domain 3
tds.crosswind*(isScalingSystemDomain==0)	4	Spatial	Domain 3

2.4.4. No Flux 1

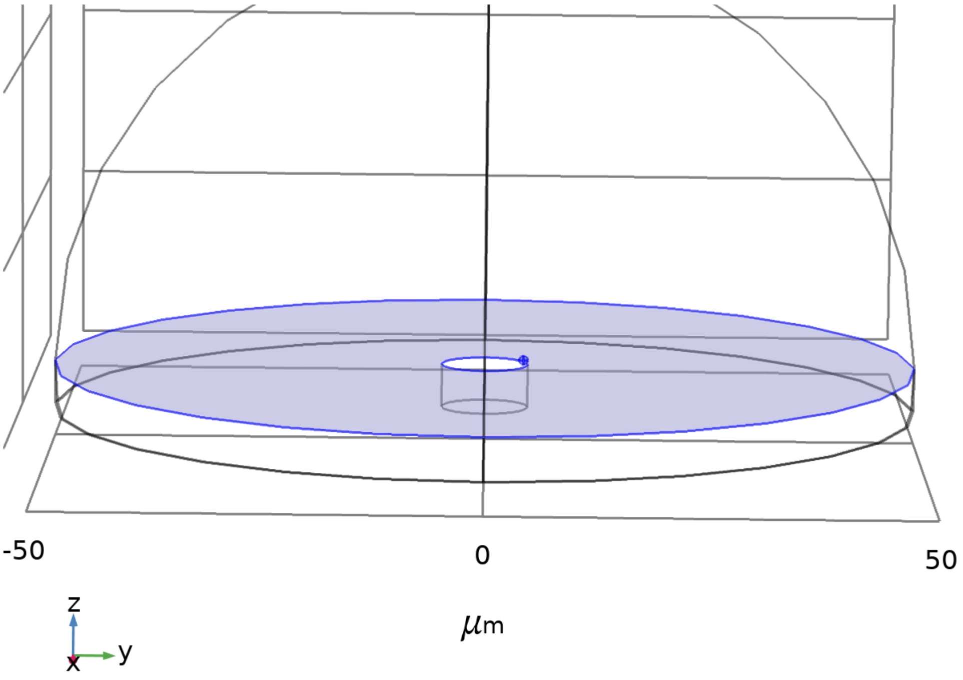

No Flux 1

SELECTION

Geometric entity level	Boundary
Selection	Geometry geom1: Dimension 2: All boundaries

EQUATIONS

$$-\mathbf{n}\cdot\mathbf{J}_i = 0$$

Convection

SETTINGS

**Description**	**Value**
Include	Off

2.4.5. Initial Values 1

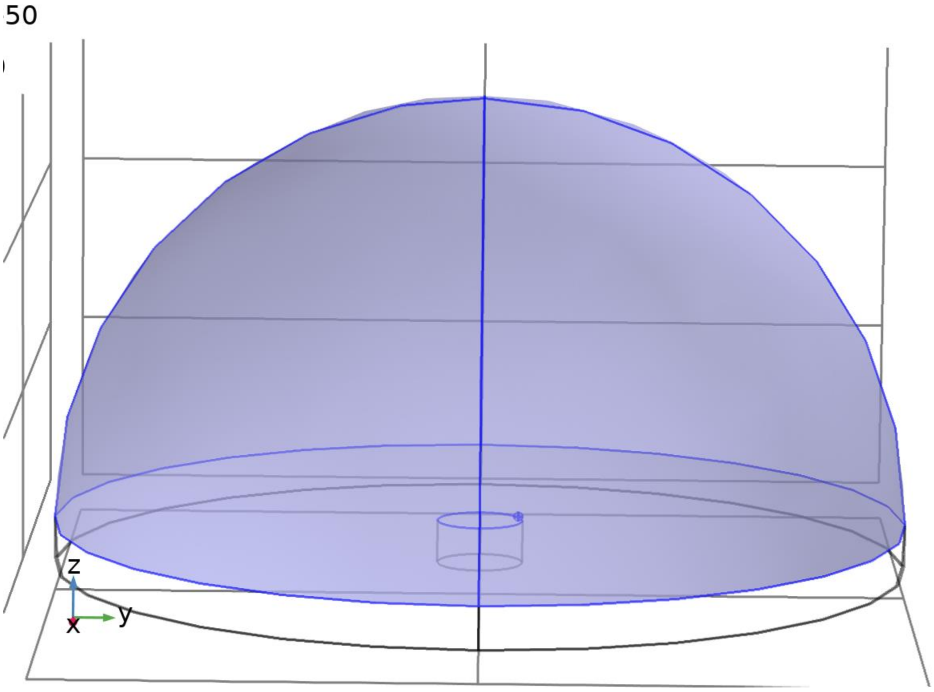

Initial Values 1

SELECTION

Geometric entity level	Domain
Selection	Geometry geom1: Dimension 3: All domains

Initial values

SETTINGS

**Description**	**Value**
Concentration	0

2.4.6. Concentration 1

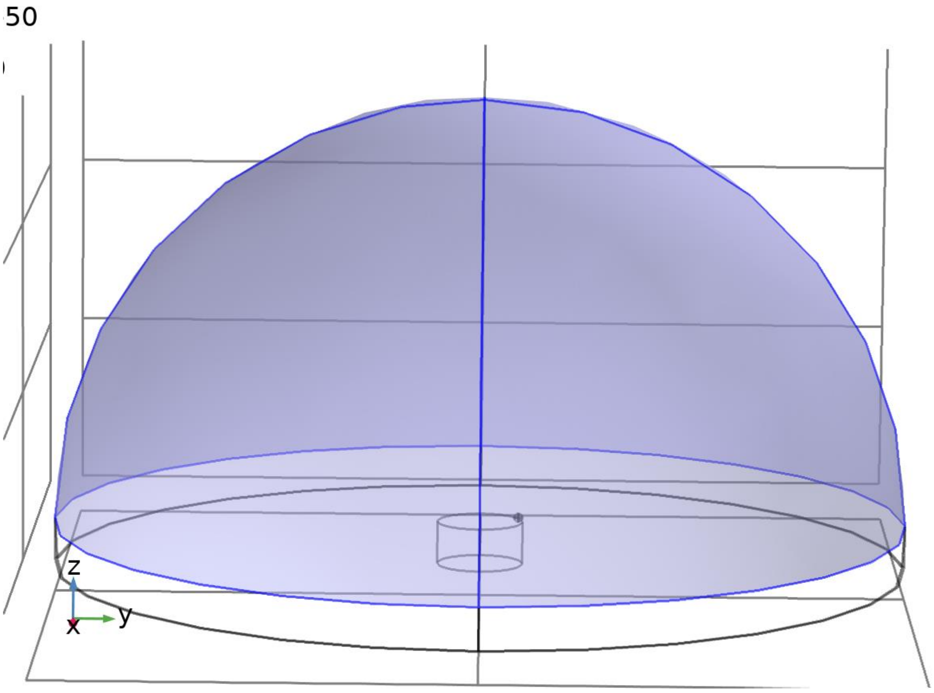

Concentration 1

SELECTION

Geometric entity level	Boundary
Selection	Geometry geom1: Dimension 2: Boundaries 6, 8, 20, 22

EQUATIONS

$$c_i = c_{0,j}$$

Concentration

SETTINGS

Description	Value
Species c	On
Concentration	c0

Variables

Name	Expression	Unit	Description	Selection
tds.c0_c	c0	mol/m^3	Concentration	Boundaries 6, 8, 20, 22

Constraints

Constraint	Constraint force	Shape function	Selection	Details
-c+tds.c0_c	test(-c+tds.c0_c)	Lagrange (Linear)	Boundaries 6, 8, 20, 22	Elemental

2.4.7. Concentration 2

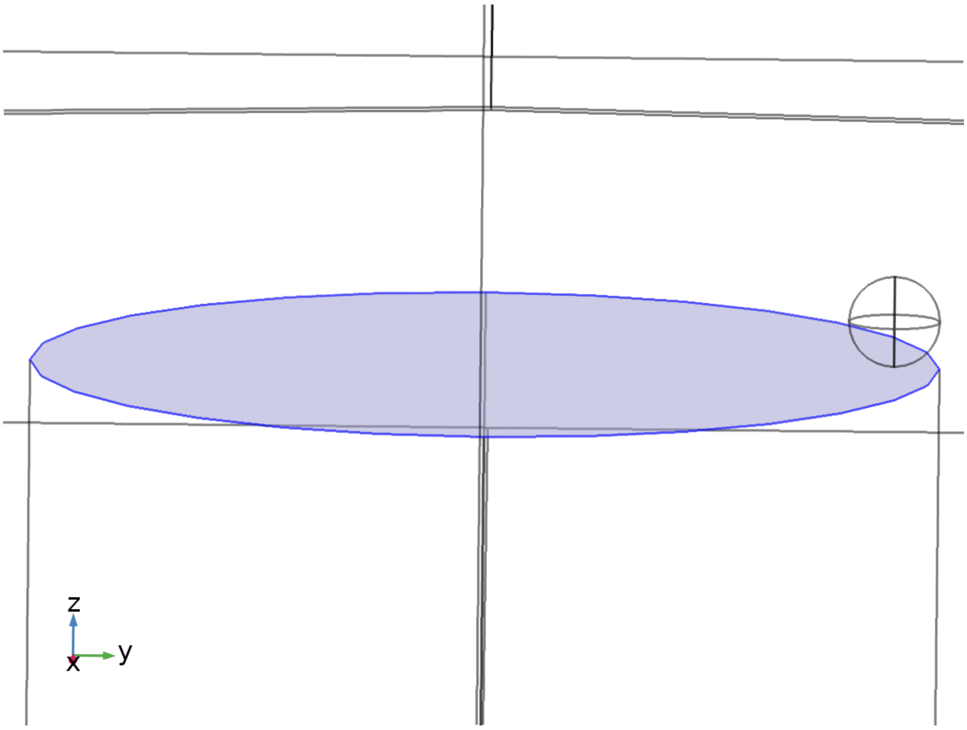

Concentration 2

SELECTION

Geometric entity level	Boundary
Selection	Geometry geom1: Dimension 2: Boundary 13

EQUATIONS

$$c_i = c_{0,j}$$

Concentration

SETTINGS

Description	Value
Species c	On
Concentration	0

Variables

Name	Expression	Unit	Description	Selection
tds.c0_c	0	mol/m³	Concentration	Boundary 13

Constraints

Constraint	Constraint force	Shape function	Selection	Details

-c+tds.c0_c	test(-c+tds.c0_c)	Lagrange (Linear)	Boundary 13	Elemental

2.5. MESH 1

MESH STATISTICS

Description	Value
Minimum element quality	0.228
Average element quality	0.6718
Tetrahedron	3311447
Triangle	354386
Edge element	1824
Vertex element	15

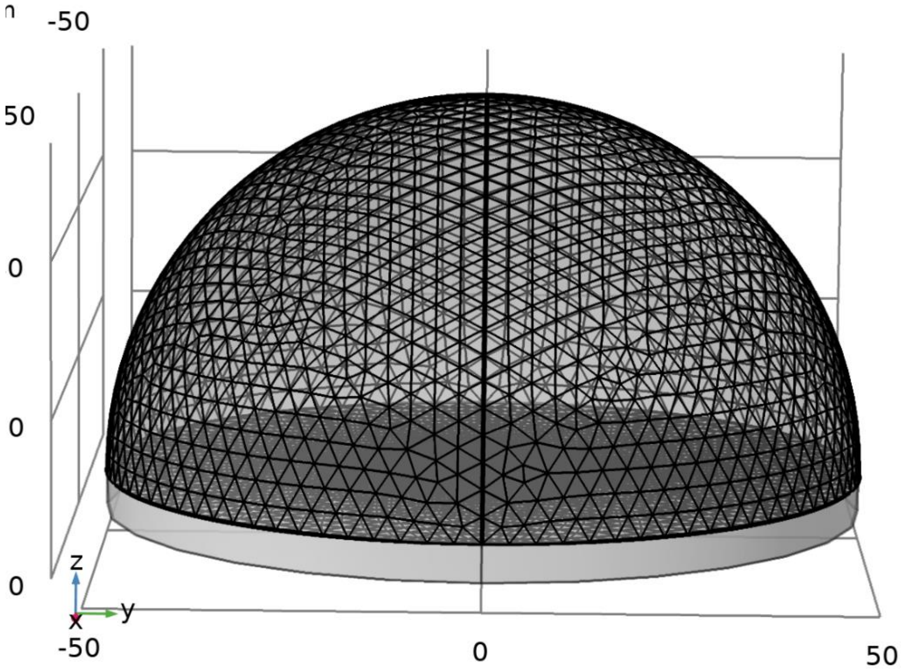

Mesh 1

2.5.1. Size (size)

SETTINGS

Description	Value
Maximum element size	3.5
Minimum element size	0.15

Curvature factor	0.3
Resolution of narrow regions	0.85
Maximum element growth rate	1.35
Predefined size	Extra fine

2.5.2. Free Tetrahedral 2 (ftet2)

SELECTION

Geometric entity level	Domain
Selection	Geometry geom1: Dimension 3: Domain 3

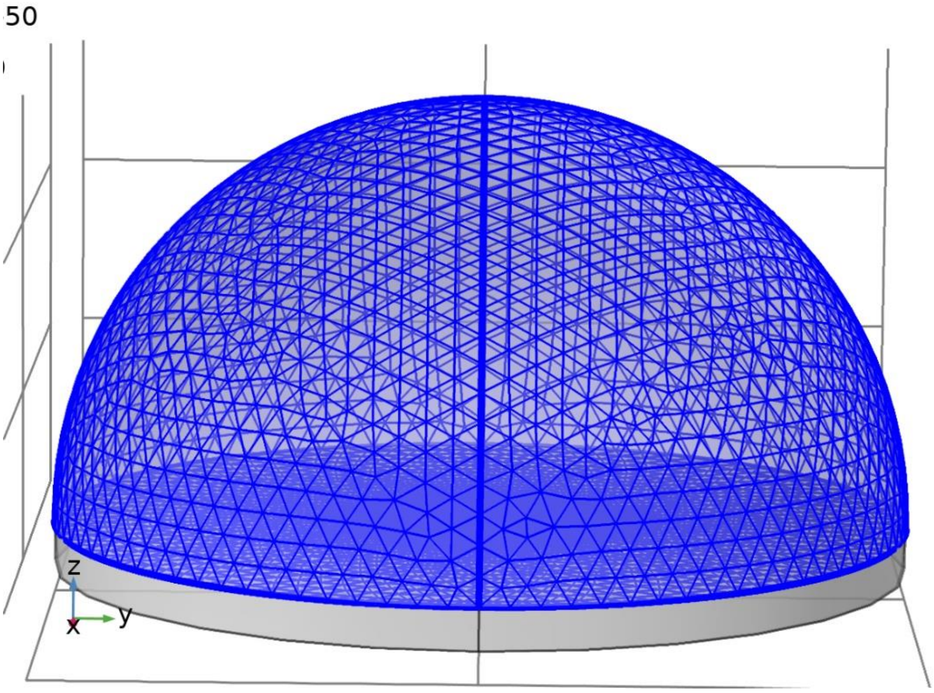

Free Tetrahedral 2

Size 1 (size1)

SELECTION

Geometric entity level	Boundary
Selection	Geometry geom1: Dimension 2: Boundaries 13–17, 23–26

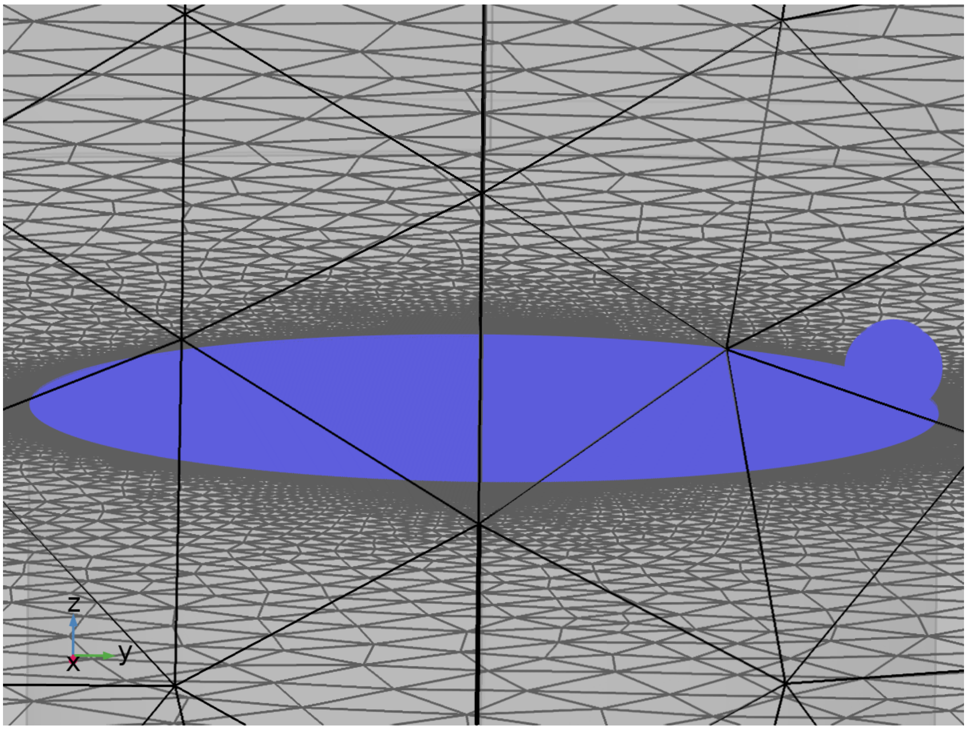

Size 1

SETTINGS

Description	Value
Maximum element size	0.025
Minimum element size	1.8
Minimum element size	Off
Curvature factor	0.6
Curvature factor	Off
Resolution of narrow regions	0.5
Resolution of narrow regions	Off
Custom element size	Custom

3. Study 1

COMPUTATION INFORMATION

Computation time	1 min 4 s
CPU	Intel64 Family 6 Model 60 Stepping 3, 4 cores

Operating system	Windows 10

3.1. STATIONARY

STUDY SETTINGS

Description	Value
Include geometric nonlinearity	Off

PHYSICS AND VARIABLES SELECTION

Physics interface	Discretization
Transport of Diluted Species (tds)	physics

MESH SELECTION

Geometry	Mesh
Geometry 1 (geom1)	mesh1

3.2. SOLVER CONFIGURATIONS

3.2.1. Solution 1

Compile Equations: Stationary (st1)

STUDY AND STEP

Description	Value
Use study	Study 1
Use study step	Stationary

LOG

```
<---- Compile Equations: Stationary in Study 1/Solution 1 (sol1) ---------------
Started at Jan 17, 2022 8:05:26 PM.
Running on Intel64 Family 6 Model 60 Stepping 3, GenuineIntel.
Using 1 socket with 4 cores in total on UTWKS60474.
Available memory: 16.29 GB.
Number of vertex elements: 15
Number of edge elements: 916
Number of boundary elements: 92938
Number of elements: 431615
Free meshing time: 19.41s
Minimum element quality: 0.1878
Geometry shape order: Linear
Time: 28 s.
Physical memory: 2.3 GB
Virtual memory: 2.53 GB
Ended at Jan 17, 2022 8:05:53 PM.
----- Compile Equations: Stationary in Study 1/Solution 1 (sol1) -------------->
```

Dependent Variables 1 (v1)

GENERAL

Description	Value
Defined by study step	Stationary

LOG

```
<---- Dependent Variables 1 in Study 1/Solution 1 (sol1) -----------------------
Started at Jan 17, 2022 8:05:53 PM.
Solution time: 1 s.
Physical memory: 1.92 GB
Virtual memory: 2.08 GB
Ended at Jan 17, 2022 8:05:54 PM.
----- Dependent Variables 1 in Study 1/Solution 1 (sol1) ---------------------->
```

Concentration (comp1.c) (comp1_c)

GENERAL

Description	Value
Field components	comp1.c
Internal variables	{comp1.uflux.c, comp1.dflux.c}

Stationary Solver 1 (s1)

GENERAL

Description	Value
Defined by study step	Stationary

LOG

```
<---- Stationary Solver 1 in Study 1/Solution 1 (sol1) ------------------------
Started at Jan 17, 2022 8:05:54 PM.
Linear solver
Number of degrees of freedom solved for: 640126 (plus 179030 internal DOFs).
Symmetric matrices found.
Format not changed since SOR line uses nonsymmetric storage.
Scales for dependent variables:
Concentration (comp1.c): 1
Orthonormal null-space function used.
Iter      SolEst     Damping    Stepsize #Res #Jac #Sol LinIt   LinErr   LinRes
   1        0.78   1.0000000        0.78    1    1    1     4  8.2e-05  6.1e-06
Solution time: 35 s.
Physical memory: 2.87 GB
Virtual memory: 3.32 GB
Ended at Jan 17, 2022 8:06:30 PM.
----- Stationary Solver 1 in Study 1/Solution 1 (sol1) ----------------------->
```

Fully Coupled 1 (fc1)

GENERAL

Description	Value
Linear solver	Iterative 1

METHOD AND TERMINATION

Description	Value

Initial damping factor	0.01
Minimum damping factor	1.0E-6
Maximum number of iterations	50

Iterative 1 (i1)

GENERAL

Description	Value
Nonlinear-based error norm	On
Maximum number of iterations	400

ERROR

Description	Value
Factor in error estimate	40

Multigrid 1 (mg1)

GENERAL

Description	Value
Use hierarchy in geometries	Geometry 1

Presmoother (pr)

SOR Line 1 (sl1)

MAIN

Description	Value
Relaxation factor	0.2

SECONDARY

Description	Value
Relaxation factor	0.4

Postsmoother (po)

SOR Line 1 (sl1)

MAIN

Description	Value

Relaxation factor	0.2

SECONDARY

Description	Value
Number of secondary iterations	2
Relaxation factor	0.4

Coarse Solver (cs)

Direct 1 (d1)

GENERAL

Description	Value
Solver	PARDISO
Pivoting perturbation	1.0E-13

4. Results

4.1. DATA SETS

4.1.1. Study 1/Solution 1

SOLUTION

Description	Value
Solution	Solution 1
Component	Save Point Geometry 1

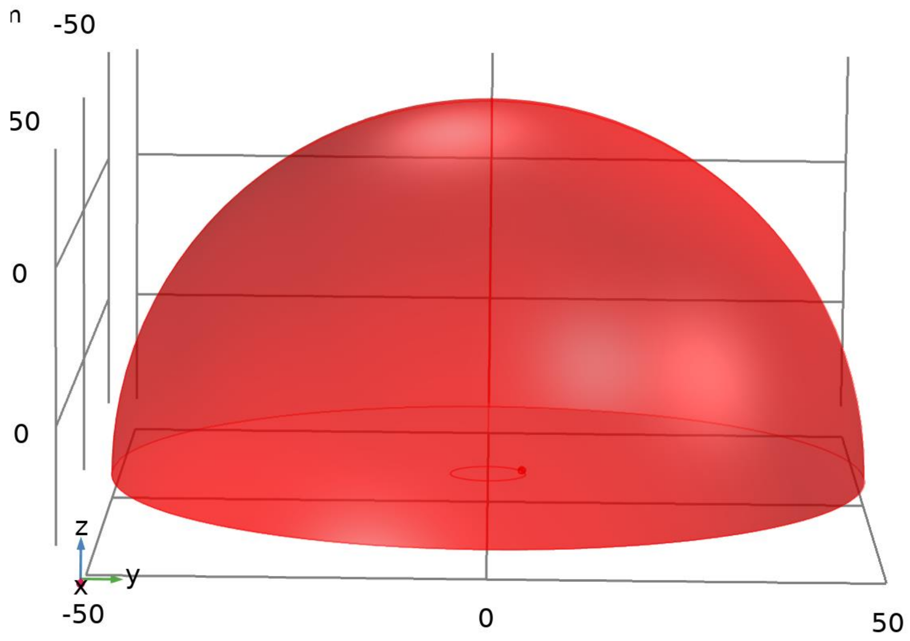

Dataset: Study 1/Solution 1

4.1.2. Cut Plane 1

DATA

Description	Value
Dataset	Study 1/Solution 1

PLANE DATA

Description	Value
Plane type	Quick
X-coordinate	0

ADVANCED

Description	Value
Space variables	{cpl1x, cpl1y}
Normal variables	{cpl1nx, cpl1ny, cpl1nz}

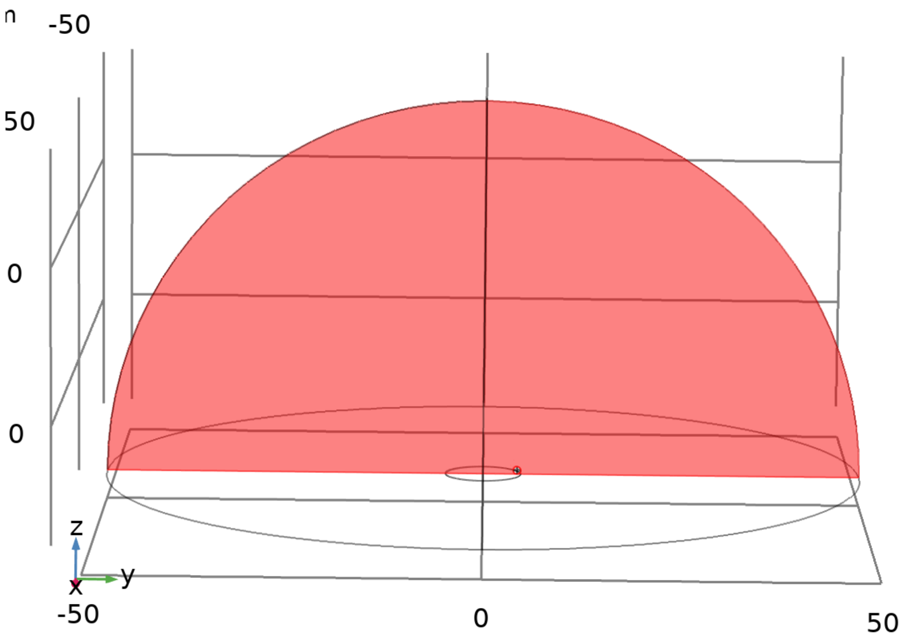

Dataset: Cut Plane 1

4.1.3. Cut Line 3D 1

DATA

Description	Value
Dataset	Study 1/Solution 1

LINE DATA

Description	Value
Line entry method	Two points
Points	{{0, -6, 5.008}, {0, 6, 5.008}}

ADVANCED

Description	Value
Space variable	cln1x

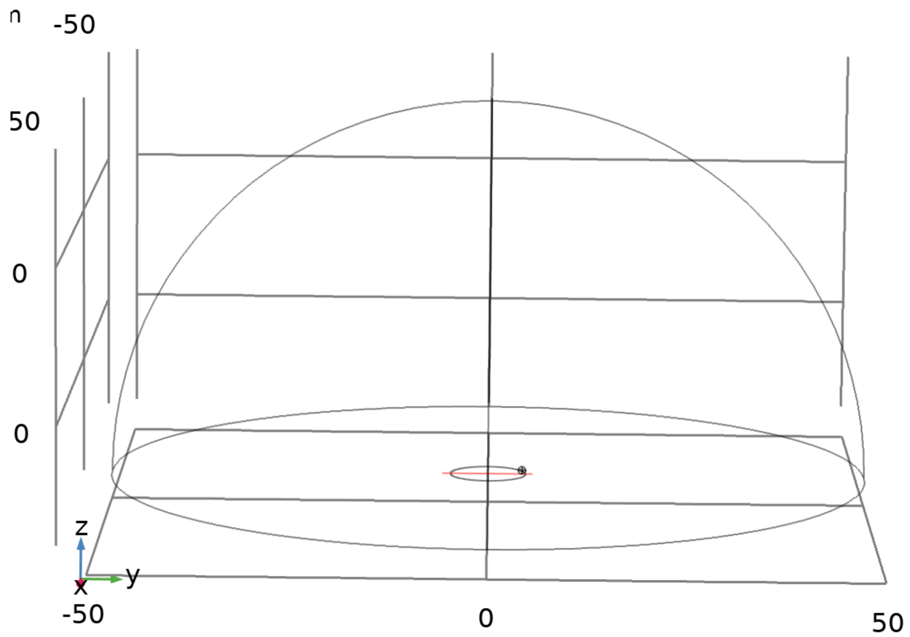

Dataset: Cut Line 3D 1

4.2. DERIVED VALUES

4.2.1. Surface Integration 1

OUTPUT

Evaluated in	Table 1

DATA

Description	Value
Dataset	Study 1/Solution 1

EXPRESSIONS

Expression	Unit	Description
D*F*tds.grad_cz	A	

INTEGRATION SETTINGS

Description	Value
Integration order	4

4.3. TABLES

4.3.1. Table 1

Surface Integration 1 (D*F*tds.grad_cz)

D*F*tds.grad_cz (A)	D*F*tds.grad_cz (A)	D*F*tds.grad_cz (A)	D*F*tds.grad_cz (A)	D*F*tds.grad_cz (A)	D*F*tds.grad_cz (A)
8.7177E-10	8.7164E-10	8.7207E-10	8.7214E-10	8.7173E-10	8.7188E-10

4.4. PLOT GROUPS

4.4.1. Concentration (tds) 1

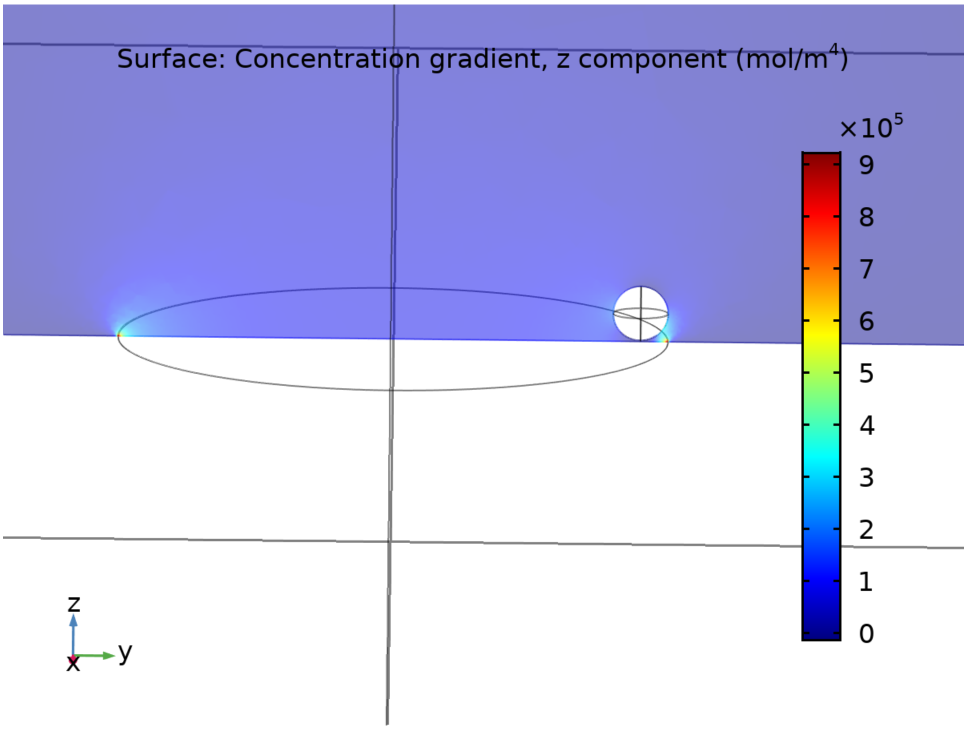

Surface: Concentration gradient, z component (mol/m^4)

4.4.2. 1D Plot Group 3

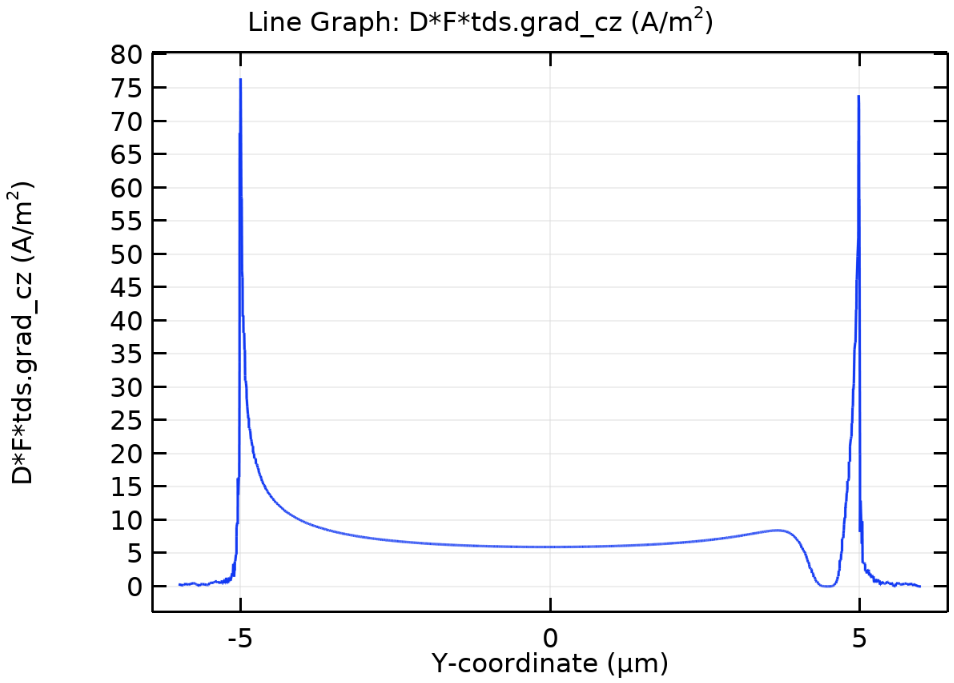

*Line Graph: D*F*tds.grad_cz (A/m^2)*
